# Telemedicine interventions after colorectal surgery: A systematic review and meta‐analysis

**DOI:** 10.1111/codi.70647

**Published:** 2026-10-11

**Authors:** Tamara I. Gimon, Garrett Johnson, Evangelia Theodosopoulos, Eleni Philippopoulos, Selina Schmocker, Marko Simunovic, Mantaj S. Brar, Erin Kennedy

**Affiliations:** ^1^ Division of General Surgery Cumming School of Medicine, University of Calgary Calgary Alberta Canada; ^2^ Division of General Surgery, Department of Surgery Mount Sinai Hospital, University of Toronto Toronto Ontario Canada; ^3^ Department of Colorectal and General Surgery Hôpital de la Cité‐de‐la‐Santé, CISSS de Laval Laval Quebec Canada; ^4^ Schulich Library of Physical Sciences, Life Sciences, and Engineering, McGill University Montreal Quebec Canada; ^5^ Division of General Surgery, Department of Surgery Juravinski Hospital Cancer Centre and McMaster University Hamilton Ontario Canada; ^6^ Zane Cohen Centre for Digestive Diseases, Mount Sinai Hospital Toronto Ontario Canada

**Keywords:** colorectal surgery, telemedicine, virtual follow‐up

## Abstract

**Aim:**

There has been increasing interest in telemedicine follow‐up after surgery. This study aims to synthesize the literature regarding the effect of postoperative telemedicine after colorectal surgery on 30‐day readmission and emergency department visit rates compared with usual care.

**Method:**

Relevant databases were searched until September 12, 2025. Two authors independently identified trials, extracted data, assessed risk of bias (ROB 2.0 and ROBINS‐I), and evidence certainty (GRADE). Randomized controlled trials (RCTs) and controlled observational studies were included. Meta‐analyses were conducted using random‐effects models. Primary outcomes were 30‐day readmissions and 30‐day emergency department visits. Secondary outcomes included patient satisfaction and compliance with the telemedicine intervention.

**Results:**

Thirteen studies (five RCTs, eight cohort studies) were included. Compared to usual care, telemedicine resulted in no difference in 30‐day readmissions (odds ratio [OR]; 0.96; 95% CI: 0.69, 1.35, 11 studies, 7102 patients, low certainty) and 30‐day emergency department visits (OR; 1.02; 95% CI: 0.67, 1.57, 8 studies, 6694 patients, low certainty). Evidence certainty was limited by serious imprecision and moderate to high risk of bias. Patient compliance with interventions ranged from 48.6% to 95.3% across studies. Patient satisfaction was higher for telemedicine compared to usual care (standardized mean difference; 0.88; 95% CI: 0.31, 1.45, 3 studies, 217 patients).

**Conclusion:**

Telemedicine interventions may lead to little difference in readmissions or emergency department visits after colorectal surgery. Telemedicine may improve patient satisfaction versus usual care. Evidence was of low certainty, indicating a need for larger randomized trials and high‐quality observational studies with appropriate design and analysis to minimize confounding.

**What does this paper add to the literature?**

This systematic review with meta‐analysis summarizes the current state of evidence for telemedicine use after colorectal surgery. Based on current evidence (13 studies, 7102 patients), telemedicine is associated with enhanced patient satisfaction, but with no differences in 30‐day readmissions or emergency department visits compared with usual care.

## INTRODUCTION

Readmission and emergency department visits are common after colorectal surgery and can result in poor health care experience, patient stress and substantial costs [1]. Emergency department visits occur in 17%–20% of colorectal surgery patients, while unplanned readmissions occur in 8%–12% [1, 2, 3, 4, 5] and may be as high as 20% in patients following ileostomy creation [6]. There has been increasing interest in developing interventions in the post‐discharge period to help decrease hospital visits after surgery, such as scheduling early office follow‐up with the patient's surgeon or primary care physician [7, 8]. However, these additional visits can be inconvenient for well patients and are subject to resource and time constraints. Telemedicine interventions in the post‐operative period have gained recent interest due to their purported cost effectiveness and patient satisfaction [9]. Platforms that have been used for postoperative telemedicine include smartphone applications [10, 11], telephone calls [12, 13], email [14] and tele‐videoconferencing [15]. These virtual applications allow for patient communication and assessment as needed from their surgical team remotely, providing more personalized medicine and potentially preventing unnecessary visits to the hospital. However, there is limited evidence in the literature exploring the effects of telemedicine virtual follow‐up after colorectal surgery.

Therefore, this systematic review and meta‐analysis aimed to synthesize the evidence on the effect of post‐discharge telemedicine and e‐health interventions after colorectal surgery on 30‐day readmission and 30‐day emergency department visit rates compared with usual follow‐up care. The secondary aim was to examine different postoperative telemedicine intervention participation rates and to evaluate their effects on patient satisfaction.

## METHODS

A systematic review and meta‐analysis was reported in accordance with the Preferred Reporting Items for Systematic Reviews and Meta‐Analyses (PRISMA) 2020 guidelines [16]. The protocol was registered in advance with the International Prospective Register of Systematic Reviews (PROSPERO), registration number: CRD42023404139. Randomized controlled trials (RCTs) and controlled observational studies were included that evaluated the effect of postoperative and post‐discharge telemedicine on adult patients (≥18 years) after colorectal surgery. Only published studies in full text form were included. Uncontrolled observational studies, reports with insufficient data, unpublished data, studies in abstract‐only form, and duplicate reports were excluded.

Colorectal surgeries included in this review were surgeries on the colon and/or rectum, stoma‐related procedures, and surgery primarily for benign perianal disease. Other general abdominal surgical procedures were excluded. For studies in which a mixture of included and excluded patient populations were presented (e.g. paediatric and adult patients), ≥90% of the analysable population had to be from the included population in the analysis.

The study intervention was defined as a telemedicine or e‐health intervention implemented in the post‐discharge period in addition to usual care follow‐up. Any studies where the only intervention was replacing in‐person follow‐up with telemedicine follow‐up were excluded. Acceptable telemedicine platforms included: mobile applications, electronic messaging, phone calls, video calls and emails. Telehealth monitoring (e.g. stoma output monitoring) was acceptable if the intervention had the ability to provide tailored advice because of the monitoring. The intervention could also include a communication feature with a healthcare provider (i.e. nurse, physician, physician assistant), or if no communication feature, had to have the ability to provide tailored advice for the patient (i.e. questionnaire, educational material). The intervention could have an active component before and during hospitalization, as long as there was a post‐discharge component after colorectal surgery (i.e. could not exclusively have a preoperative, or in‐hospital component). The comparison arm consisted of usual postoperative care delivered either contemporaneously or historically before implementation of the telemedicine intervention. Included telemedicine interventions supplemented rather than replaced standard postoperative care. Studies in which telemedicine replaced routine postoperative follow‐up were excluded. As usual postoperative care was determined by local institutional practice, some variation between studies was expected. The two primary outcomes were 30‐day readmissions and 30‐day emergency department visits. Secondary outcomes were patient satisfaction, and patient compliance with the intervention.

The following databases were searched from inception to September 12, 2025: Medline (Ovid), PubMed (non‐Medline records), Embase (Ovid), CINHAL (EBSCOhost), Cochrane Central Database of Controlled Trials (Ovid), Web of Science (Clarivate) and Google Scholar (first 100 results extracted). Relevant keywords, text words and MeSH terms relating to telemedicine and colorectal surgery were identified and searched in Medline and then translated to the other databases. No search limits pertaining to language, age, date and study type were applied in the databases. The full search strategy is available in Table S1. References of relevant full text articles and systematic reviews were assessed for any additional eligible studies. Clinicaltrials.gov and PROSPERO were searched to identify any relevant ongoing studies.

Two reviewers (TG and GJ or ET) independently screened all titles and abstracts, assessed full‐text articles for study eligibility and inclusion, and extracted data using standardized pilot‐tested data extraction sheets. Two independent reviewers also assessed risk of bias for all primary outcomes using methods suggested in the Cochrane Handbook for Systematic Reviews of Interventions [17]. The revised Cochrane risk‐of‐bias tool for randomized trials (RoB 2) was used for RCTs, and the ROBINS‐I V2 tool for observational studies [18, 19]. Overall evidence certainty for each primary outcome was evaluated according to the GRADE approach [20], using GRADEpro GDT software (https://www.gradepro.org/). Discrepancies in screening, extraction, and analysis were resolved by discussion, and if needed by a third reviewer (EK).

Odds ratio (OR) estimates were calculated for 30‐day readmissions and 30‐day emergency department visits comparing those who received a telemedicine intervention with usual care. OR were calculated by pooling individual study estimates, weighted using the DerSimonian and Laird method, and analysed with a random‐effects model. When available, adjusted estimates rather than unadjusted estimates were used and extracted as absolute numbers. Standardized mean difference (SMD) were calculated for patient satisfaction rates as multiple scales were used to measure the same outcome. We conducted data synthesis in Review Manager 5.4 for all study outcomes using both narrative and quantitative methods. For studies where more than one comparison was possible (i.e. multiple arms meeting inclusion criteria), we selected the condition most similar to the other comparator interventions in the analysis. Where a study contained unclear information, such as missing details to inform risk of bias assessments, details of interventions and outcomes, and study results, the study corresponding author was contacted to ensure accuracy, and to retrieve missing data.

Publication biases were assessed by viewing study confidence interval overlap. Analysis using the funnel plot technique was planned for instances when at least 10 studies were included in the review for a particular outcome [21]. We assessed statistical heterogeneity through visual inspection using forest plots, and via standard *χ* ^2^ statistic and the associated *I* ^2^ statistic [22]. We considered heterogeneity to be substantial when the *χ* ^2^ statistic was significant at the *p* = 0.1 level, or when the *I* ^2^ statistic suggested that more than 40% of the variability in effect estimate was due to heterogeneity [22].

Subgroup and sensitivity analyses were conducted to investigate heterogeneity. Subgroup analysis was planned to assess differences in outcome between RCTs versus observational studies, between mobile application versus other telemedicine platforms, and timing of telemedicine intervention (pre‐ and postoperative vs. postoperative only). Sensitivity analyses for the primary outcomes were planned to examine the effect of risk of bias, by excluding studies deemed at high risk; by changing from random‐effects to fixed‐effect models; and by changing from OR to risk ratio (RR) and risk difference (RD). Odds ratios (ORs) were selected as the primary effect measure because they are the standard metric for pooling dichotomous outcomes across randomized and observational studies and are consistent with adjusted estimates reported in several included observational studies.

## RESULTS

A PRISMA flow diagram is presented in Figure 1. The database search and manual reference review identified 2276 citations. After 1172 duplicates were removed, 1104 abstracts were screened, of which 1039 studies did not meet the inclusion criteria. Sixty‐five studies went on to full‐text review. Thirteen studies were included in our final systematic review and meta‐analysis [10, 11, 13, 14, 23, 24, 25, 26, 27, 28, 29, 30, 31]. Eight records (seven RCTs, one observational study) for potentially included ongoing studies were identified [32, 33, 34, 35, 36, 37, 38, 39]. Forty‐four were excluded. Detailed rationale for studies excluded at full‐text review and details of the ongoing studies are available in Tables S2 and S3, respectively.

**FIGURE 1:**
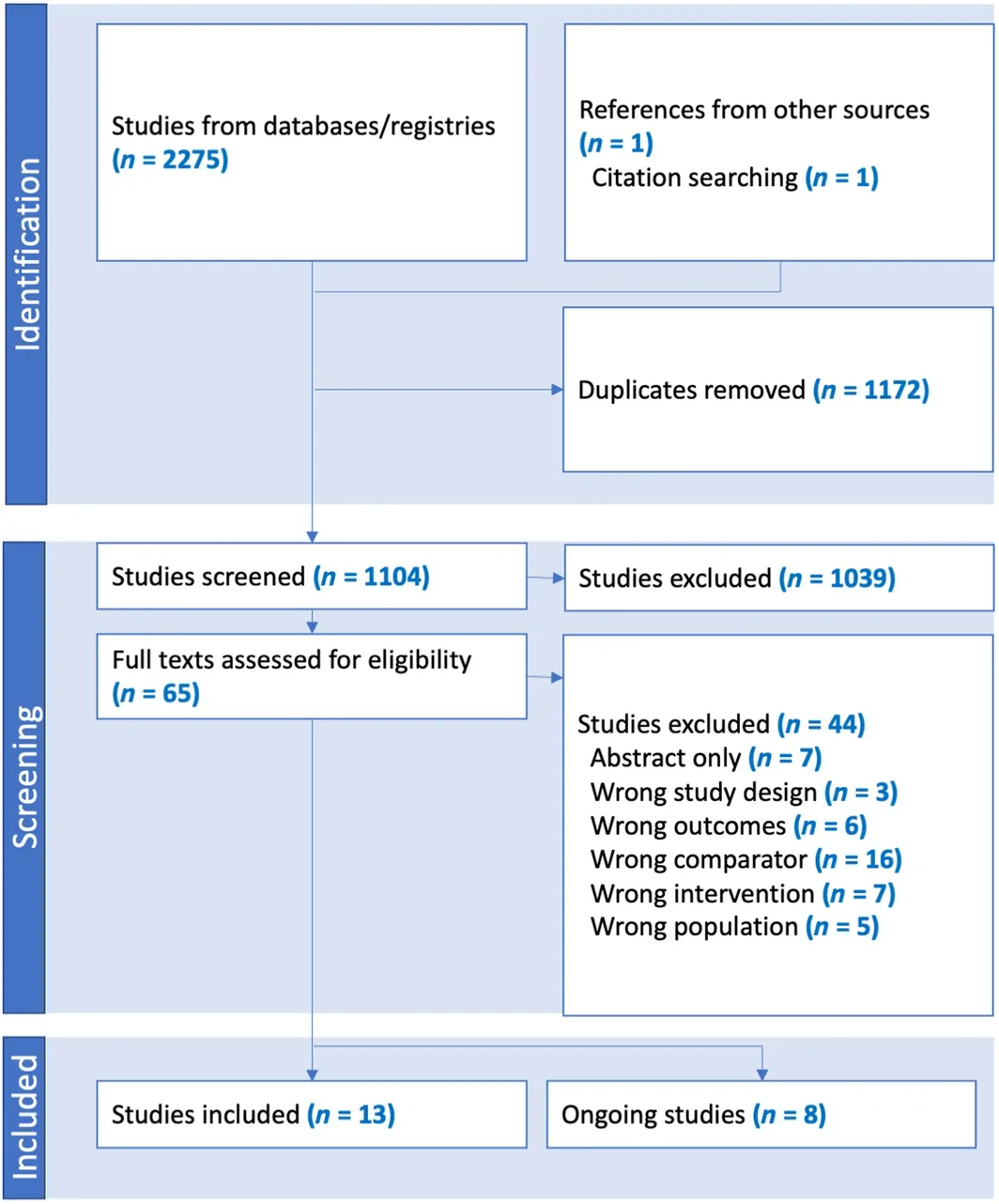
PRISMA flow diagram.

Studies are summarized in Table 1. Studies were conducted in Australia [13], the Netherlands [29], Canada [10, 11, 28], the United States of America [14, 23, 24, 25, 26], China [30, 31], and Taiwan [27]. Publication year ranged from 2013 to 2025 with 11 of 13 being published since 2020 [10, 11, 14, 24, 25, 26, 27, 28, 29, 30, 31]. Nine studies included only elective surgery [10, 11, 13, 14, 23, 24, 26, 28, 29], one study included both elective and emergency colorectal surgeries [25], and in three studies surgery urgency was unclear [27, 30, 31]. One study included only rectal cancers [30], the remainder included a mixture of abdominopelvic colorectal operations. Two studies included primarily patients with inflammatory bowel disease and therefore had a substantially younger population [10, 14], compared to other studies with mixed colorectal surgery patients [11, 23, 24, 25, 26, 29], or cancer [13, 27, 30, 31].

**TABLE 1:** Characteristics of included studies on postoperative telemedicine interventions after colorectal surgery.

Study ID	Design	Intervention type	Detailed description	2‐way communication	Response rates	Patient satisfaction	Intervention population
Borsuk 2019 [23] USA	Retrospective cohort (281 patients)	Mobile app (Twistle)	Available pre and postoperatively Secure patient–provider messaging Preoperative automated patient education alerts (e.g., chlorhexidine wipes day before surgery) Postoperative daily symptom questionnaires with alerts to providers if concerning response (or no response) Wound photo upload capability	Patients, physicians, physicians' office staff, nurses, care coordinators, and extended care nurses could all communicate via the app	73.2% enrolled and interacted with the app at least once	Not reported	Mean age 60.3 years. Mixed colorectal patients (proportions NOS)
Eustache 2023 [11] Canad^a^	Ambi‐directional cohort (prospective intervention, retrospective control; 350 patients)	Mobile app (CareSense)	Available pre and postoperatively Secure patient‐provider messaging Digital version of usual ERAS patient education materials accessible pre‐ and postoperatively Postoperative symptom questionnaires with alerts to providers if concerning responses Positive wound responses triggered requests for wound photos	Patients could message their care team, with responses provided by the surgeon or residents (monitored 7 AM–5 PM daily.)	55% ‘used the app’	Likelihood of recommending app to ‘another patient undergoing surgery’ (Mean 10‐point Likert scale; intervention group only): 9.4, SD 1.5	Mean age 56.1 years. Mixed colorectal patients (72.8% cancer, 14.9% IBD, 12.3% other)
Gleason 2022 [24] USA	Retrospective cohort (158 patients)	Mobile app (SeamlessMD)	Available pre and postoperatively Healthcare reminder alerts, and access to digital educational content and resources pre‐ and postoperatively Postoperative daily health checks and patient‐reported outcome surveys, send alerts to clinical nursing staff	Daily health checks send alerts to clinical nursing staff. No information how providers would respond to alerts	48.6% ‘used the app’	Yes/No Questions (intervention group only): ‘Helped feel prepared for surgery’ 91.7%; ‘Helped take care of self at home after surgery’ 89.6%; ‘Would recommend to others’ 95.8%	Mean age 58.4 years. Mixed colorectal patients (proportions NOS)
Huber 2025 [26] USA	Retrospective cohort (3147 patients)	Emails and web‐based portal (MyMSK)	Available postoperatively only Daily survey to monitor patients' recovery/symptoms, with surgical team notified if concerning responses	Very concerning symptoms notify on‐call surgical team to respond. Intermediate symptoms notify surgical nurse to call during regular business hours	70.2% responded to at least one of the surveys	Not reported	Median age 59 years Mixed colorectal patients (proportions NOS)
Li 2024 [30] China	Retrospective cohort (72 patients)	Mobile app and web‐based portal (WeChat)	Available postoperatively only Personalized discharge plan based on patient needs identified in hospital Patient‐provider messaging (24‐h online nursing care), with ability to request in‐person care/assessment Nurses can upload documentation and patient wound photos from home visits	24‐h patient to nurse messaging with optional in person assessment	Not reported	Total mean MES‐OCE scores: Intervention: 142.31, SD 8.83 Control: 130.56, SD 6.50	Mean age: 58.98. Rectal cancer only
Liao 2023 [27] Taiwan	Parallel group, open label RCT (pilot/feasibility study; 107 patients)	Mobile app and wristband fitness tracker (Xiaomi wristband mi band 2)	Available pre and postoperatively Device records patient step counts and reports them to their surgical team	Research assistant would contact participant if they did not upload step count data for more than 7 days	72.6% submitted step count per week (average across weeks)	Not reported	Mean age: 58.87 years. Colorectal cancer only
Lu 2024 [31] China	Parallel group, open label RCT (92 patients)	Mobile app and web‐based portal (WeChat)	Available postoperatively only Patient‐provider messaging Educational materials targeted towards patient caregivers (i.e., family) regarding disease education, rehabilitation, precautions, and methods to deal with stress of being a caregiver	Patients could comment publicly on resources. Doctors and nurses would moderate and address issues. Participants also received the private contact for nurses or doctor to contact them with problems	Not reported	Mean self‐reported satisfaction at 4 weeks (10‐point Likert scale): Intervention 7.5, SD 1.4 Control: 6.8, SD 1.7	Mean age: 73.7 years. Colorectal cancer only
Paradis 2023 [28] Canada^a^	Ambi‐directional cohort (prospective intervention, retrospective control; 114 patients)	Mobile app (CareSense)	Available pre and postoperatively Secure patient‐provider messaging Digital version of usual ERAS patient education materials accessible pre‐ and postoperatively Postoperative symptom questionnaires with alerts to providers if concerning responses Positive wound responses triggered requests for wound photos	Patients could message their care team, with responses provided by the surgeon or residents (monitored 7 AM–5 PM daily)	76.9% in the ‘high patient activation’ group	Not reported	Mean age: 57.2 years. Mixed colorectal (66.7% cancer, 11.1% IBD, 20.5% other)
Pooni 2023 [10] Canada	Parallel group, open label RCT (253 patients)	Mobile app and emails (Home to Stay)	Available postoperatively only Educational materials on self‐management and recovery Daily health checks/symptom surveys (email) with automated tailored responses and escalation to care team as appropriate until POD 30. Wound photo upload capability	Responses to daily health checks were monitored by two PAs from 8 AM to 2 PM and escalated to the surgeon for phone follow‐up as necessary	57.4% Completed at least one health check	‘Satisfaction with the discharge process’ (Median 10‐point Likert scale): Intervention: 9, IQR: 8–10. Control: 8, IQR: 7–9	Mean age: 41.0 years. Mixed colorectal (18.0% cancer, 68.0% IBD, 14.0% other)
Stapler 2022 [25] USA	Retrospective cohort (1720 patients)	Mobile app	Available pre and postoperatively Secure patient‐provider messaging Preoperative automated patient education alerts (e.g., exercise, bowel preparation) Postoperative symptom questionnaires with automated recommendations and alerts to care providers if concerning responses	Messages monitored by the nurse navigator during the day and on‐call physician at night and on weekends	Not reported	Not reported	Mean age: 61.7 years. Mixed colorectal (25.4% cancer. 9.7% IBD, 64.9% other)
van der Storm 2025 [29] Netherlands	Parallel group, open label RCT (140 patients)	Mobile app (APPtimize)	Available pre and postoperatively Pre and postoperative automated patient education reminders/alerts targeted at ERAS pathway recommendations Daily activity monitoring (step counter), and ERAS item compliance sent to research team, and care providers	No provider communication. Contact research team for technical support only	Not reported	Median satisfaction (Scale from 2 to 10) Intervention: 8 Control: 8	Mean age: 55 years. Mixed colorectal (56.9% cancer, 11.1% IBD, 32% other)
Young 2013 [13] Australia	Parallel group, open label RCT (704 patients)	Phone calls (Connect program)	Available postoperatively only Scheduled nurse‐led phone calls using standardized screening protocols and address patient needs Escalation to local providers for high‐risk problems outside of study nurse scope	Structured nurse phone calls with escalation to other practitioners as appropriate	83.7%–96% answered calls (varied by time interval)	Not reported	Mean age 68.6 years. Colorectal cancer only
Zand 2020 [14] USA	Retrospective cohort (128 patients)	Emails	Available postoperatively only Direct e‐mail access to a specialized, surgical IBD nurse for questions Structured questionnaires and prompts for wound photos Daily nurse monitoring, and escalation to surgeon as appropriate	Patients had direct e‐mail access to a specialized, surgical IBD nurse for questions. The nurse would also check survey responses and escalate to the surgeon as appropriate	95.3% sent ≥1 wound photo	‘Experience participating in the post surgery questionnaire and follow‐up program’: ‘Excellent’: 81.3%, ‘Good’: 12.5%, ‘Had no effect on recovery’: 6.3%	Mean age 37.9 years. IBD only

Abbreviations: COI, conflicts of interest; IBD, inflammatory bowel disease; IQR, interquartile range; MES‐OCE, Medical Experience Scale for Outpatient Care of Enterostomy; NOS, not otherwise specified; POD, postoperative day; RCT, randomized controlled trial; SD, standard deviation; USA, United States of America.

^a^ Uses the same intervention at the same centre according to two separate cohorts of patients.

There were five RCTs [10, 13, 27, 29, 31], and eight observational studies: six retrospective cohort [14, 23, 24, 25, 26, 30] and two ambi‐directional cohort studies [11, 28]. All studies were unblinded. Three studies' authors declared potential conflicts of interest due to industry relationships [11, 28, 29], although no studies were industry sponsored.

Eleven studies used a mobile telemedicine application [10, 11, 23, 24, 25, 26, 27, 28, 29, 30, 31], one used phone calls [13], and one used email questionnaires [14]. In seven studies, patients were invited to utilize the telemedicine intervention preoperatively [11, 23, 24, 25, 27, 28, 29], which included preoperative education in addition to postoperative components. Nine applications used two‐way communication, where the participant could interact with their care team [10, 11, 13, 14, 23, 25, 28, 30, 31]. In two applications, the care team would only interact with the participant if concerns were identified [24, 26].

Assessment of readmissions and emergency department visits varied across studies. Six studies identified emergency department visits and/or readmissions from chart data at the study site, but not external hospitals [11, 14, 23, 25, 26, 28]. Two studies further stratified hospital visit reasons as avoidable versus unavoidable based upon patient diagnoses [11, 23]. One study extracted readmission rates from the American College of Surgeons National Surgical Quality Improvement Program (ACS‐NSQIP) database [24]. Two studies extracted readmissions and emergency department visit rates based on patient report, rather than relying on administrative data [10, 13]. In two studies, the source of visit data was not specified, so it is unclear if admission to external hospitals would have been captured [27, 29].

### 30‐day readmissions

There was no significant difference between telemedicine and usual care for the primary outcome of 30‐day readmissions (OR: 0.96, 95% CI: 0.69, 1.35, 11 studies, 7102 participants, *I* ^2^ = 67%, low certainty evidence; Figure 2). There was substantial heterogeneity, which can be explained by study design, as the RCT subgroup had minimal heterogeneity (*I* ^2^ = 0%). The overall risk of bias was assessed as low in two RCTs [10, 13], and high in two others [27, 29]. Three observational studies were at moderate overall risk of bias [11, 25, 26], and four were at serious risk of bias [14, 23, 24, 28]. Risk of bias assessments are included in Table S4, in the supplement. Accordingly, evidence certainty for this outcome was limited due to serious risk of bias, and serious imprecision (wide confidence intervals that cross one).

**FIGURE 2:**
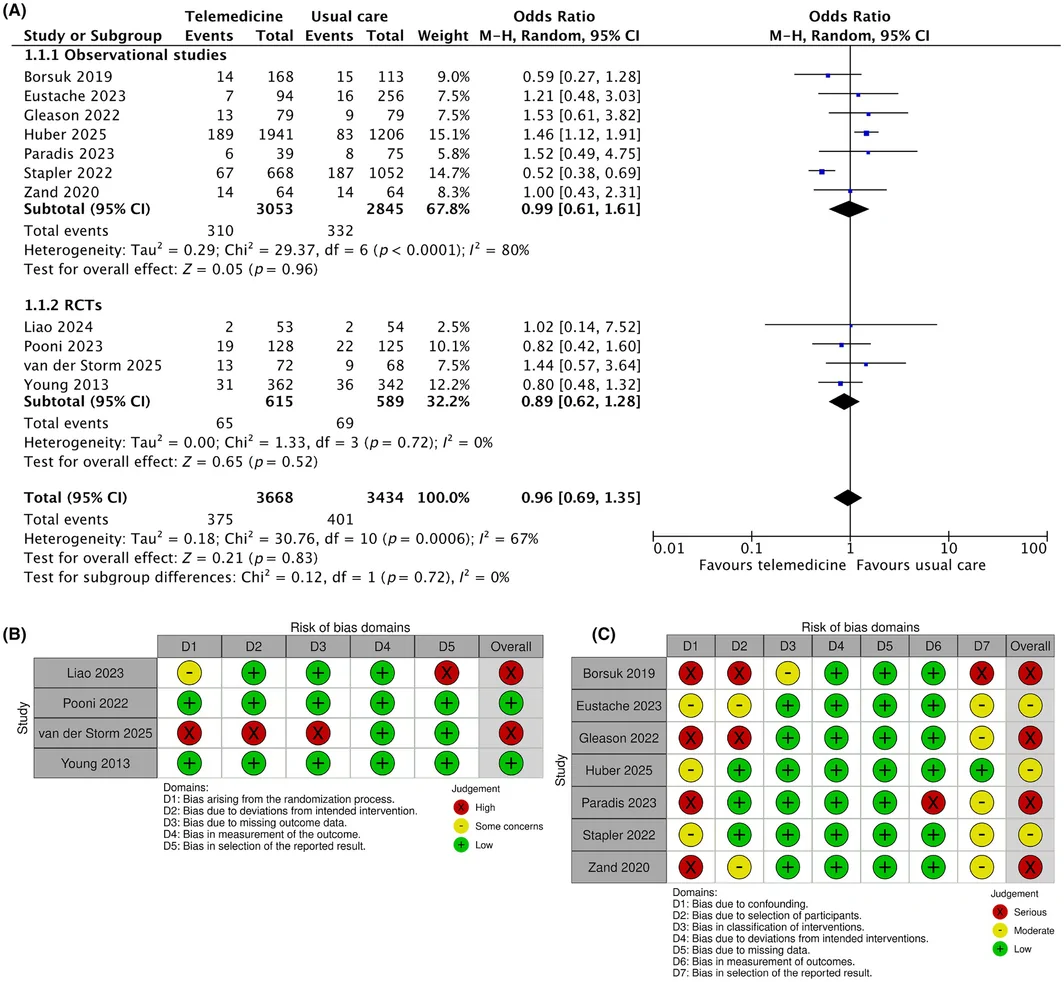
Effect of telemedicine interventions compared to usual care on 30‐day readmission after colorectal surgery stratified by study design: (A) Forest plot and stop light diagrams for risk of bias assessment using (B) ROB 2.0 tool for randomized controlled trials and (C) ROBINS‐I tool for observational studies.

Subgroup (Figures S1 and S2) and sensitivity analyses (Figures S3–S6), and summary of findings with Grade assessment (Table S5) are available in the supplement. There remained no significant difference in 30‐day readmissions between groups on planned subgroup and sensitivity analysis that included stratifying results by modality (mobile application vs. other) and timing of telemedicine intervention (postoperative only vs. pre‐ and postoperative), and analysis according to fixed effects model, assessment of risk ratio, risk difference, and exclusion of studies at severe risk of bias. The funnel plot for this outcome was symmetric, suggesting no publication bias (Figure S7).

### 30‐day emergency department visits

There was no significant difference between groups for the primary outcome of 30‐day emergency department visits (OR: 1.02, 95% CI: 0.67, 1.57, 8 studies, 6694 participants, *I* ^2^ = 80%, low certainty evidence, Figure 3). There was substantial heterogeneity for this outcome, which was again minimal in the RCT subgroup (*I* ^2^ = 0%). Both included RCTs were assessed as low risk of bias for this outcome [10, 13]. Three observational studies were at moderate risk of bias [11, 25, 26] and three were at serious risk of bias [14, 23, 28]. Evidence certainty for this outcome was limited due to serious risk of bias and serious imprecision. There was no difference between groups on planned subgroup (Figures S8 and S9) and sensitivity analysis (Figures S10–S12).

**FIGURE 3:**
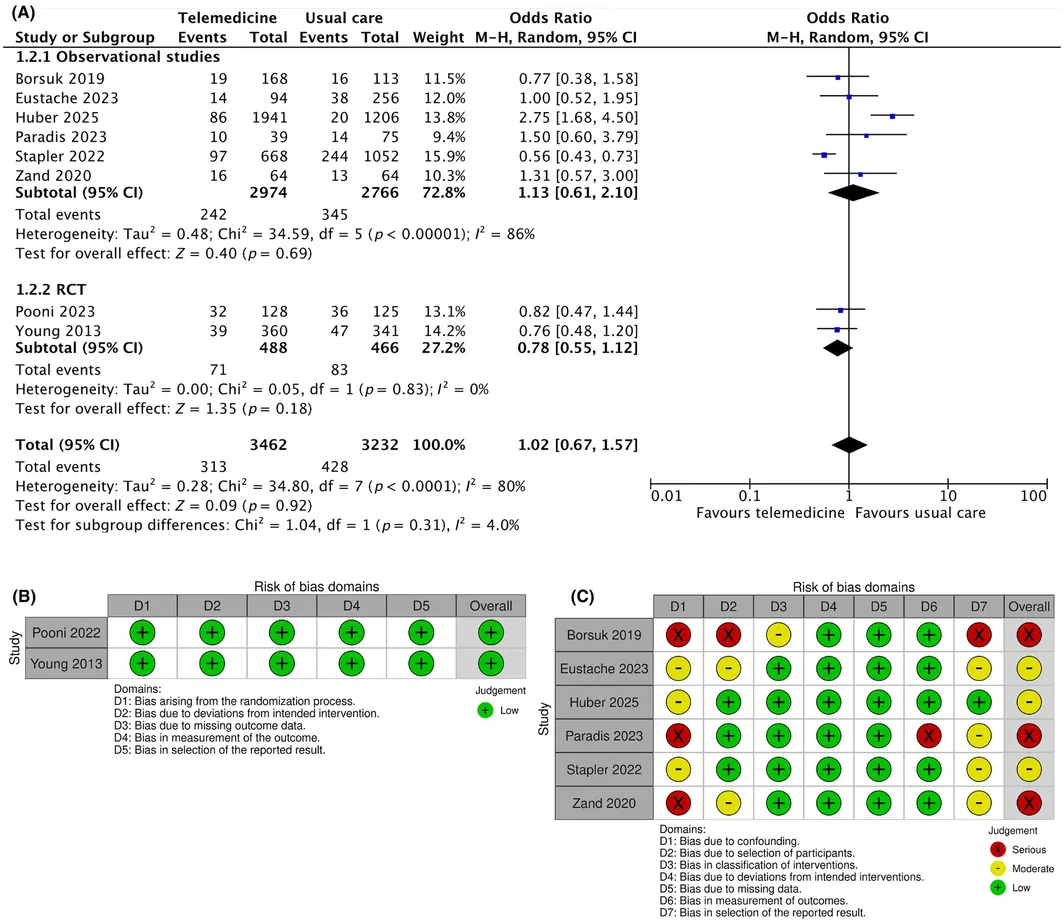
Effect of telemedicine interventions compared to usual care on 30‐day emergency department visits after colorectal surgery stratified by study design: (A) Forest plot and stop light diagrams for risk of bias assessment using (B) ROB 2.0 tool for RCTs and (C) ROBINS‐I tool for observation studies.

### Patient satisfaction

Patient satisfaction was reported in seven studies, all using different assessment tools. Four studies compared satisfaction between intervention and control arms [10, 29, 30, 31]. Five studies used a variation on a 10‐point Likert scale [10, 11, 29, 30, 31]. Only one study used a previously validated assessment scale [30]. One study assessed patient satisfaction with the program via multiple choice questionnaire with options ‘no effect’, ‘good’ or ‘excellent’ [14]. Finally, one study assessed satisfaction using yes/no questions [24].

Patient satisfaction was significantly higher for patients assigned to telemedicine compared to usual care (SMD: 0.75, 95% CI: 0.55, 0.95, 3 studies, 417 participants, *I* ^2^ = 84%; Figure 4). Sensitivity analysis including a fourth trial by imputing missing standard deviation data [29], did not substantially affect results' magnitude or statistical significance (Figures S13–S16). On sensitivity analysis, when the non‐randomized study assessing this outcome was excluded [30], results did not change, but heterogeneity was minimized (*I* ^2^ = 0%, Figure S17).

**FIGURE 4:**
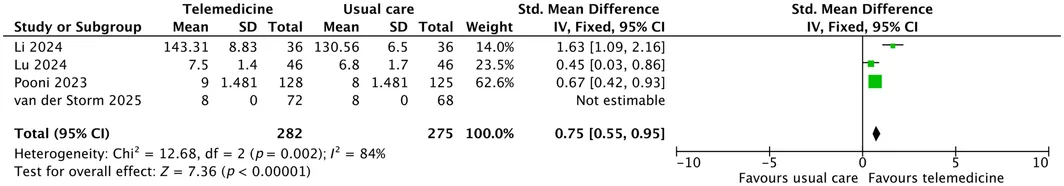
Forest plot on the effect of telemedicine interventions compared to usual care on patient satisfaction.

### Telemedicine participation rate

Participation with the telemedicine intervention varied between 48.6% and 95.3% across studies. The two studies that did not use a mobile application had the highest patient participation rates (83.7%–95.3%) [13, 14]. There was no apparent relationship between participation rate and patient age, indication for surgery, or whether the application had a two‐way communication feature (Table 1).

## DISCUSSION

The currently available evidence suggests that telemedicine interventions may have little to no effect on 30‐day readmissions or emergency department visits after colorectal surgery; however, confidence in these estimates is limited by clinical and methodological heterogeneity and the overall low certainty of the available evidence. Participation rates varied considerably, and were highest with telephone calls and email as opposed to mobile applications. While patient satisfaction was higher among patients who received telemedicine interventions, less than half of the studies reported patient satisfaction as an outcome and only one study used a validated scale [30].

The results of our systematic review are similar to the results of other systematic reviews assessing the effectiveness of telemedicine to support patients in the post‐discharge setting, which have shown mixed effects on 30‐day readmission rates and emergency visits. A recent systematic review and meta‐analysis examining postoperative telemedicine interventions among a breadth of surgical specialties found no benefit for emergency department visits (RR: 0.89, 95% CI: 0.70, 1.12, 18 studies) and readmissions (RR: 0.90, 95% CI: 0.74–1.09, 22 studies) [40]. The authors' conclusions were that any true benefit may have been diluted by the heterogeneity of surgical specialties and procedures included in the review, thus calling for more specific analyses examining specific interventions and surgical specialties. Conversely, another systematic review examining cellphone or tablet‐based mobile applications (excluding other forms of telemedicine) after surgery identified a modest reduction in both emergency department visits (OR: 0.42, 95% CI: 0.23, 0.79, 4 studies) and readmissions (OR: 0.47, 95% CI: 0.29, 0.77, 6 studies) across surgical specialties and disease sites. This latter review was limited by including a much smaller number of studies for each outcome [41]. These differences in results may reflect incomplete study capture in both reviews. Despite overlapping inclusion criteria, each review included studies that appeared to meet the other's criteria but were not identified by the authors.

The effectiveness of telemedicine interventions also varies between individual included studies. One reason for these inconsistent findings is likely the lack of high‐quality RCTs and the inclusion of observational studies with potential residual confounding and selection bias. In several observational studies, patients receiving telemedicine may have differed systematically from those receiving usual care. For example, those selected for telemedicine may have been perceived to be at higher postoperative risk or greater need for follow‐up. Although some studies adjusted for measured confounders, residual confounding cannot be excluded and contributed to the overall low certainty of the evidence. Notably, despite the potential for selection bias in the observational studies, pooled estimates were similar between randomized and observational studies, suggesting that any resulting bias did not substantially alter the overall findings. Clinical heterogeneity between studies may also have contributed to study variability, as the review included patients undergoing colorectal surgery for different indications with varying postoperative care needs. Additionally, usual postoperative care likely differed across institutions with respect to follow‐up schedules, access to nursing support, and enhanced recovery pathways, which may have influenced the incremental benefit observed with telemedicine interventions.

Another factor is that telemedicine appropriately identifies patients requiring urgent assessment. In this circumstance, telemedicine could increase emergency department visits or readmissions by facilitating earlier recognition of postoperative complications, while simultaneously improving patient outcomes. Consequently, the absolute number of hospital visits may not fully capture the value of postoperative telemedicine. Future studies should evaluate the appropriateness of emergency department visits and readmissions, major postoperative complications, failure‐to‐rescue, unplanned reoperations, and mortality to better determine whether telemedicine improves the quality and safety of postoperative care. Therefore, the appropriateness of hospital utilisation, rather than the absolute number of emergency department visits or readmissions alone, may be a more meaningful measure of telemedicine effectiveness.

Another factor affecting the effectiveness of telemedicine interventions found in this review is the relatively low participation rates for use of the interventions. Low participation rates may lead to negative results if the benefits experienced by participants using the intervention are offset by the participants who do not use the app. Reasons for low and variable uptake were not evaluated in included studies, but are likely to be complex [42]. For example, patients with higher digital literacy, reliable internet access, and stronger perceived benefit may be more likely to engage. Provider endorsement and integration of telemedicine into standard discharge workflows may also influence uptake. At the system level, platform usability, privacy concerns, and reimbursement policies affect adoption. Finally, cultural perceptions on the role of technology versus in‐person visits in perioperative care would also affect uptake. Accounting for these factors during telemedicine implementation requires a thoughtful integration into clinical pathways, education, and equitable access, all with associated costs. Unfortunately, none of the studies in this review included a cost‐effectiveness assessment. This is important as increasingly these interventions are being interfaced with the electronic medical record. For example, the Epic electronic medical record system (Epic Systems Corporation, Verona, Wisconsin) has an integrated MyChart feature allowing patient‐provider secure direct communication already [43]. In future research, telemedicine cost‐effectiveness studies should evaluate costs of the implementation strategy in addition to the costs of the platform itself.

Given the inconsistent benefits of telemedicine for reducing readmissions and emergency visits, it may be that the main benefit of telemedicine after colorectal surgery is enhancing the patient experience, likely stemming from these interventions leading to increased patient contact with the health care system in ‘real time’ when they need it most. Patient satisfaction was reported in the minority of studies in this review, and only one used a validated scale. For future research, the Quality of Recovery Scale is a validated scale that incorporates patient‐reported outcomes (including patient satisfaction) into their postoperative assessment [44]. This Quality of Recovery Scale has been used as the primary outcome in two previous high‐quality RCTs with over 1000 patients in which use of a mobile app was found to significantly improve quality of recovery after non‐colorectal surgery and these improved scores were associated with cost savings [45, 46].

The current evidence base suggests that a definitive evaluation of postoperative telemedicine following colorectal surgery remains needed. Future randomized trials should enrol homogeneous surgical populations (e.g., colorectal cancer, inflammatory bowel disease, or elective colorectal surgery separately), use standardized telemedicine interventions with clearly described components, and incorporate implementation outcomes such as patient adherence and intervention fidelity. Importantly, future studies should evaluate outcomes beyond hospital utilization alone. While emergency department visits and readmissions remain important measures of healthcare resource use, they may not adequately capture the effectiveness of telemedicine if earlier identification of postoperative complications appropriately increases healthcare utilization while preventing more severe adverse events. Accordingly, future trials should include measures of the appropriateness of hospital utilization, major postoperative complications, failure‐to‐rescue, reoperation, patient‐reported outcomes using validated instruments, and cost‐effectiveness. Adequately powered multicentre randomized trials incorporating these outcomes will be necessary to determine the true clinical value of postoperative telemedicine following colorectal surgery.

### Limitations

The main limitation of this review is the lack of high‐quality RCTs and the differing types of telemedicine interventions used, leading to low overall evidence certainty. Currently, there are eight ongoing studies identified (seven RCTs), which have the potential to change the conclusions of this review when completed. Another limitation is the clinical heterogeneity of the included populations. Studies enrolled patients undergoing colorectal surgery for a range of indications, including colorectal cancer, inflammatory bowel disease, benign colorectal disease, and mixed colorectal cohorts. While the mechanisms through which postoperative telemedicine may influence recovery are likely similar across these populations, differences in baseline patient characteristics, postoperative complications, and follow‐up requirements may limit the applicability of the pooled estimates to individual disease‐specific populations. Future studies evaluating telemedicine within more homogeneous surgical populations would help determine whether effectiveness differs by indication. We were also not able to evaluate resource intensity or cost effectiveness, as none of the included studies evaluated this outcome.

## CONCLUSION

Current evidence does not demonstrate a reduction in emergency department visits or readmissions following colorectal surgery, although confidence in these estimates is limited by the low certainty and heterogeneity of the available evidence. Despite low and variable participation rates, patient satisfaction with use of the interventions is higher compared to usual care. Future high‐quality RCTs with outcome measures including resource intensity (i.e., ED visits and readmissions), patient‐reported outcomes and cost effectiveness are necessary to adequately evaluate the impact of these interventions in the post‐discharge setting following surgery.

## AUTHOR CONTRIBUTIONS

**Tamara I. Gimon:** Conceptualization; methodology; data curation; investigation; validation; formal analysis; writing – original draft; writing – review and editing; visualization. **Garrett Johnson:** Methodology; data curation; formal analysis; validation; investigation; visualization; writing – original draft; writing – review and editing; conceptualization; software. **Evangelia Theodosopoulos:** Investigation; writing – review and editing; data curation; conceptualization. **Eleni Philippopoulos:** Methodology; writing – review and editing; data curation; conceptualization. **Selina Schmocker:** Data curation; writing – review and editing; conceptualization; project administration. **Marko Simunovic:** Methodology; writing – review and editing; conceptualization. **Mantaj S. Brar:** Conceptualization; methodology; writing – review and editing. **Erin Kennedy:** Conceptualization; methodology; supervision; resources; project administration; writing – review and editing; writing – original draft; formal analysis.

## FUNDING INFORMATION

The authors have nothing to report.

## CONFLICT OF INTEREST STATEMENT

All authors have no relevant disclosures or competing interests to declare.

## ETHICS STATEMENT

This is a systematic review and meta‐analysis of published literature and no ethics approval was sought.

## Supporting information


Data S1

## Data Availability

The data that supports the findings of this study are available in the supplementary material of this article.

## References

[1] Wick EC , Shore AD , Hirose K , Ibrahim AM , Gearhart SL , Efron J , et al. Readmission rates and cost following colorectal surgery. Dis Colon Rectum. 2011;54:1475–1479. 10.1097/DCR.0b013e31822ff8f0 22067174

[2] O'Connell EP , Healy V , Fitzpatrick F , Higgins CA , Burke JP , McNamara D . Predictors of readmission following proctectomy for rectal cancer. Dis Colon Rectum. 2019;62:703–710. 10.1097/DCR.0000000000001355 30762598

[3] Wood T , Aarts M‐A , Okrainec A , Pearsall E , Victor JC , McKenzie M , et al. Emergency room visits and readmissions following implementation of an enhanced recovery after surgery (iERAS) program. J Gastrointest Surg. 2018;22:259–266. 10.1007/s11605-017-3555-2 28916971

[4] Li LT , Mills WL , White DL , Li A , Gutierrez AM , Berger DH , et al. Causes and prevalence of unplanned readmissions after colorectal surgery: a systematic review and meta‐analysis. J Am Geriatr Soc. 2013;61:1175–1181. 10.1111/jgs.12307 23730901

[5] Eustache J , Hopkins B , Trepanier M , Kaneva P , Fiore JF Jr , Fried GM , et al. High incidence of potentially preventable emergency department visits after major elective colorectal surgery. Surg Endosc. 2022;36:2653–2660. 10.1007/s00464-021-08514-x 33959806

[6] Vogel I , Shinkwin M , van der Storm SL , Torkington J , A.Cornish J , Tanis PJ , et al. Overall readmissions and readmissions related to dehydration after creation of an ileostomy: a systematic review and meta‐analysis. Tech Coloproctol. 2022;26:333–349. 10.1007/s10151-022-02580-6 PMC9018644 35192122

[7] Lumpkin ST , Strassle PD , Fine JP , Carey TS , Stitzenberg KB . Early follow‐up after colorectal surgery reduces Postdischarge emergency department visits. Dis Colon Rectum. 2020;63:1550–1558. 10.1097/DCR.0000000000001732 PMC9554947 33044296

[8] Moneme AN , Wirtalla CJ , Roberts SE , Keele LJ , Kelz RR . Primary care physician follow‐up and 30‐day readmission after emergency general surgery admissions. JAMA Surg. 2023;158:1293–1301. 10.1001/jamasurg.2023.4534 PMC10534988 37755816

[9] Asiri A , AlBishi S , AlMadani W , ElMetwally A , Househ M . The use of telemedicine in surgical care: a systematic review. Acta Inform Med. 2018;26:201–206. 10.5455/aim.2018.26.201-206 PMC6195401 30515013

[10] Pooni A , Brar MS , Anpalagan T , Schmocker S , Rashid S , Goldstein R , et al. Home to stay: a randomized controlled trial evaluating the effect of a postdischarge mobile app to reduce 30‐day readmission following elective colorectal surgery. Ann Surg. 2023;277:e1056–e1062. 10.1097/SLA.0000000000005527 35815882

[11] Eustache JH , Latimer EA , Liberman AS , Charlebois P , Stein BL , Fiore JF , et al. A Mobile phone app improves patient‐physician communication and reduces emergency department visits after colorectal surgery. Dis Colon Rectum. 2023;66:130–137. 10.1097/DCR.0000000000002187 34933314

[12] Iqbal A , Raza A , Huang E , Goldstein L , Hughes SJ , Tan SA . Cost effectiveness of a novel attempt to reduce readmission after ileostomy creation. JSLS. 2017;21:e2016.00082. 10.4293/JSLS.2016.00082 PMC5266511 28144122

[13] Young JM , Butow PN , Walsh J , Durcinoska I , Dobbins TA , Rodwell L , et al. Multicenter randomized trial of centralized nurse‐led telephone‐based care coordination to improve outcomes after surgical resection for colorectal cancer: the CONNECT intervention. J Clin Oncol. 2013;31:3585–3591. 10.1200/JCO.2012.48.1036 24002519

[14] Zand A , Nguyen A , Stokes Z , van Deen W , Lightner A , Platt A , et al. Patient experiences and outcomes of a telehealth clinical care pathway for postoperative inflammatory bowel disease patients. Telemed J E Health. 2020;26:889–897. 10.1089/tmj.2019.0102 31670610

[15] Bednarski BK , Nickerson TP , You YN , Messick CA , Speer B , Gottumukkala V , et al. Randomized clinical trial of accelerated enhanced recovery after minimally invasive colorectal cancer surgery (RecoverMI trial). Br J Surg. 2019;106:1311–1318. 10.1002/bjs.11223 PMC7938853 31216065

[16] Page MJ , McKenzie JE , Bossuyt PM , Boutron I , Hoffmann TC , Mulrow CD , et al. The PRISMA 2020 statement: an updated guideline for reporting systematic reviews. Int J Surg. 2021;88:105906. 10.1016/j.ijsu.2021.105906 33789826

[17] Higgins JPT , Savović J , Page MJ , Elbers RG , Sterne JAC . Assessing risk of bias in a randomized trial. In: Higgins JP , Thomas J , Chandler J , Cumpston M , Li T , Page MJ , et al., editors. Cochrane handbook for systematic reviews of interventions. London: Cochrane. cited [2025 Aug 13]. Available from: https://www.cochrane.org/authors/handbooks‐and‐manuals/handbook/current/chapter‐08; 2019.

[18] Higgins JPT , Sterne J , Savović J , Page MJ , Hróbjartsson A , Boutron I , et al. A revised tool for assessing risk of bias in randomized trials. Cochrane Database Syst Rev. 2016;10(Suppl 1):29–31.

[19] Sterne J , Higgins J . ROBINS‐I V2 tool. 2024 [cited 2025 Aug 19]. Available from: https://sites.google.com/site/riskofbiastool/welcome/robins‐i‐v2

[20] The GRADE Working Group . GRADE handbook for grading quality of evidence and strength of recommendations; 2013. Available from: https://www.guidelinedevelopment.org/handbook. Accessed 13 August 2025.

[21] Page MJ , Higgins JPT , Sterne J . Assessing risk of bias due to missing evidence in a meta‐analysis. In: Higgins JP , Thomas J , Chandler J , Cumpston M , Li T , Page MJ , et al., editors. Cochrane Handbook for Systematic Reviews of Interventions, London: Cochrane. [cited 2025 Aug 13]. Available from: https://www.cochrane.org/authors/handbooks‐and‐manuals/handbook/current/chapter‐13 (2024).

[22] Higgins JPT , Thompson SG , Deeks JJ , Altman DG . Measuring inconsistency in meta‐analyses. BMJ. 2003;327:557–560. 10.1136/bmj.327.7414.557 PMC192859 12958120

[23] Borsuk DJ , Al‐Khamis A , Geiser AJ , Zhou D , Warner C , Kochar K , et al. S128: active post discharge surveillance program as a part of enhanced recovery after surgery protocol decreases emergency department visits and readmissions in colorectal patients. Surg Endosc. 2019;33:3816–3827. 10.1007/s00464-019-06725-x 30859488

[24] Gleason LT , Xie R , Wood L , Shao C , dos Santos Marques IC , Donahue JM , et al. Cost–benefit analysis of a patient engagement technology (PET) in cardiac, thoracic, and colorectal surgery. Am J Surg. 2022;224:979–986. 10.1016/j.amjsurg.2022.04.030 35525626

[25] Stapler S‐J , Brockhaus KK , Battaglia MA , Mahoney ST , McClure AM , Cleary RK . A single‐institution analysis of targeted colorectal surgery enhanced recovery pathway strategies that decrease readmissions. Dis Colon Rectum. 2022;65:e728–e740. 10.1097/DCR.0000000000002129 34897213

[26] Huber HM , Wei IH , Abbass MA , Karagkounis G , Widmar M , Smith JJ , et al. Impact of patient‐reported outcome monitoring via recovery tracker on post‐discharge outcomes after colorectal surgery: a comparative analysis before and after implementation. Cancer. 2025;17:1939. 10.3390/cancers17121939 PMC12191032 40563589

[27] Liao Y‐T , Chou Y‐J , Wu C‐T , Liu YH , Liang JT , Lai F , et al. Wearable mobile health device for monitoring postoperative ambulation among patients with colorectal cancer undergoing minimally invasive surgery: a prospective comparison study. Int. J. Med. Robot. Comput. Assist. Surg. 2024;20:e2626. 10.1002/rcs.2626 38517612

[28] Paradis T , Robitaille S , Dumitra T , Liberman AS , Charlebois P , Stein BL , et al. The impact of patient activation on the effectiveness of digital health remote post‐discharge follow‐up and same‐day‐discharge after elective colorectal surgery. Surg Endosc. 2024;38:1548–1555. 10.1007/s00464-023-10597-7 38114879

[29] van der Storm SL , Jansen M , Mulder MD , Marsman HA , Consten ECJ , den Boer FC , et al. Improving enhanced recovery after surgery (ERAS): the effect of a patient‐Centred Mobile application and an activity tracker on patient engagement in colorectal surgery. Surg Innov. 2025;32:5–15. 10.1177/15533506241299888 PMC11736976 39514899

[30] Li M , Yu K , Zhang Y , Mao A , Dong LY . Impact of discharge planning combined with “internet home ostomy care platform” in patients with permanent colostomy after rectal cancer surgery. Ann Ital Chir. 2024;95:699–707. 10.62713/aic.3459 39186342

[31] Lu H , Wang H , Zhu L , Xu J , Su Z , Dong W , et al. The impact of WeChat online education and care on the mental distress of caregivers and satisfaction of elderly postoperative colorectal cancer patients. Clin Res Hepatol Gastroenterol. 2024;48:102372. 10.1016/j.clinre.2024.102372 38719145

[32] Diehl TM , Barrett JR , Abbott DE , Cherney Stafford LM , Hanlon BM , Yang Q , et al. Protocol for the MobiMD trial: a randomized controlled trial to evaluate the effect of a self‐monitoring mobile app on hospital readmissions for complex surgical patients. Contemp Clin Trials. 2022;113:106658. 10.1016/j.cct.2021.106658 PMC8844087 34954099

[33] Steffens D , Koh C , Smith A , Mohan HM , Carey S , Smith SM , et al. Virtual multimodal hub for patients undergoing major gastrointestinal cancer surgery (PRIORITY‐CONNECT 2 pilot) – a pilot randomised type I hybrid effectiveness‐implementation trial. Surg Oncol Insight. 2024;1:100082. 10.1016/j.soi.2024.100082

[34] Storm M , Morken IM , Austin RC , Nordfonn O , Wathne HB , Urstad KH , et al. Evaluation of the nurse‐assisted eHealth intervention ‘eHealth@Hospital‐2‐home’ on self‐care by patients with heart failure and colorectal cancer post‐hospital discharge: protocol for a randomised controlled trial. BMC Health Serv Res. 2024;24:18. 10.1186/s12913-023-10508-5 PMC10768157 38178097

[35] NCT06540430. Digital based home care service for ostomy patients. 2024. Available from: https://clinicaltrials.gov/study/NCT06540430?term=NCT06540430&rank=1. Accessed 17 October 2025.

[36] NCT06593678. Telerehabilitation in cancer patients: optimization of prehabilitation and rehabilitation following colorectal resection (CCR). 2024. Available from: https://clinicaltrials.gov/study/NCT06593678?term=NCT06593678&rank=1. Accessed 17 October 2025.

[37] NCT06157814. Mobile ostomates resources for patients and caregivers (MORE). 2023. Available from: https://clinicaltrials.gov/study/NCT06157814?term=NCT06157814&rank=1. Accessed 17 October 2025.

[38] NCT06302751. A mobile application to improve postoperative outcomes in colorectal cancer (RAISe‐Care). 2024. Available from: https://clinicaltrials.gov/study/NCT06302751?term=NCT06302751&rank=1. Accessed 17 October 2025.

[39] NCT06850688. Impact of ambulatory continuous monitoring using remote monitoring medicine within patient's care pathway following colorectal surgery (CONTACT‐GRECCAR 21) (CONTACT). 2025. Available from: https://clinicaltrials.gov/study/NCT06850688?term=NCT06850688&rank=1. Accessed 17 October 2025.

[40] Eustache J , El‐Kefraoui C , Ekmekjian T , Latimer E , Lee L . Do postoperative telemedicine interventions with a communication feature reduce emergency department visits and readmissions?‐a systematic review and meta‐analysis. Surg Endosc. 2021;35:5889–5904. 10.1007/s00464-021-08607-7 34231068

[41] Dawes AJ , Lin AY , Varghese C , Russell MM , Lin AY . Mobile health technology for remote home monitoring after surgery: a meta‐analysis. Br J Surg. 2021;108:1304–1314. 10.1093/bjs/znab323 34661649

[42] Mahajan A , Islam SD , Schwartz MJ , Cannesson M . A hospital is not just a factory, but a complex adaptive system‐implications for perioperative care. Anesth Analg. 2017;125:333–341. 10.1213/ANE.0000000000002144 28614127

[43] Chishtie J , Sapiro N , Wiebe N , Rabatach L , Lorenzetti D , Leung AA , et al. Use of epic electronic health record system for health care research: scoping review. J Med Internet Res. 2023;25:e51003. 10.2196/51003 PMC10757236 38100185

[44] Myles PS , Shulman MA , Reilly J , Kasza J , Romero L . Measurement of quality of recovery after surgery using the 15‐item quality of recovery scale: a systematic review and meta‐analysis. Br J Anaesth. 2022;128:1029–1039. 10.1016/j.bja.2022.03.009 35430086

[45] Dahlberg K , Philipsson A , Hagberg L , Jaensson M , Hälleberg‐Nyman M , Nilsson U . Cost‐effectiveness of a systematic e‐assessed follow‐up of postoperative recovery after day surgery: a multicentre randomized trial. Br J Anaesth. 2017;119:1039–1046. 10.1093/bja/aex332 29077819

[46] Jaensson M , Dahlberg K , Eriksson M , Nilsson U . Evaluation of postoperative recovery in day surgery patients using a mobile phone application: a multicentre randomized trial. Br J Anaesth. 2017;119:1030–1038. 10.1093/bja/aex331 29077818

