# Supplementary material for: Telemedicine interventions after colorectal surgery: A systematic review and meta‐analysis
Source: Colorectal Dis. 2026 Oct 11;28(10):e70647. doi: 10.1111/codi.70647 (PMC13654625; doi:10.1111/codi.70647)
Supplement: Supplementary file 1 [file CODI-28-0-s001.docx]

**Online supplementary materials: Telemedicine after colorectal surgery**

Table of Contents

[eTable 1. Detailed search strategy	2](#_Toc217898955)

[eTable 2. Excluded studies details	9](#_Toc217898956)

[eTable 3. Detailed Characteristics of Ongoing studies	14](#_Toc217898957)

[eTable 4: Risk of bias assessments for the outcomes of 30-day readmission and 30-day emergency department visits	27](#_Toc217898958)

[eTable 5. Summary of findings	29](#_Toc217898959)

[Supplementary Forest plots	29](#_Toc217898960)

[1.	30-day readmissions	29](#_Toc217898961)

[eFigure 1. Subgroup analysis 30-day readmissions stratified by smartphone applications and analogue applications (phone calls and email).	30](#_Toc217898962)

[eFigure 2. Subgroup analysis 30-day readmission stratified by timing of telemedicine intervention	30](#_Toc217898963)

[eFigure 3. Sensitivity analysis 30-day readmission with fixed effects model	31](#_Toc217898964)

[eFigure 4. Sensitivity analysis 30-day readmission with risk ratio reported	31](#_Toc217898965)

[eFigure 5. Sensitivity analysis 30-day readmission with risk difference reported	32](#_Toc217898966)

[eFigure 6. Sensitivity analysis 30-day readmission studies at severe risk of bias excluded.	32](#_Toc217898967)

[eFigure 7. Funnel plot for 30-day readmissions stratified by trial design (randomized controlled trial; RCT versus observational studies).	33](#_Toc217898968)

[2.	30-day emergency department visits	34](#_Toc217898969)

[eFigure 8. Subgroup analysis 30-day emergency department visits stratified by smartphone applications and analogue applications (phone calls and email).	34](#_Toc217898970)

[eFigure 9. Subgroup analysis 30-day emergency department visits stratified by time of telemedicine intervention	34](#_Toc217898971)

[eFigure 10. Sensitivity analysis 30-day emergency department visits with fixed effects model	35](#_Toc217898972)

[eFigure 11. Sensitivity analysis 30-day emergency department visits with risk ratio reported	35](#_Toc217898973)

[eFigure 12. Sensitivity analysis 30-day emergency department visits with risk difference reported	36](#_Toc217898974)

[3.	Patient satisfaction	36](#_Toc217898975)

[eFigure 13. Sensitivity analysis patient satisfaction with van der Storm 2025 imputed from other studies	36](#_Toc217898976)

[eFigure 14. Sensitivity analysis patient satisfaction imputed van der Storm 2025 standard deviation imputed as a small value	36](#_Toc217898977)

[eFigure 15. Sensitivity analysis patient satisfaction with van der Storm 2025 imputed as a large value	37](#_Toc217898978)

[eFigure 16. Sensitivity analysis patient satisfaction with a fixed effects model	37](#_Toc217898979)

[eFigure 17. Sensitivity analysis patient satisfaction with only RCTs included (Li 2024 excluded)	37](#_Toc217898980)

# eTable 1. Detailed search strategy

| **ICTRP** (WHO) | search sep 12, 2025 |
| --- | --- |
| search terms | Results |
| 1 | (telemedicine OR telehealth OR teleconsultation OR telecare OR telemonitoring OR telerehabilitation OR "tele intervention" OR ehealth OR "tele medicine" OR "tele health" OR "tele communication" OR "tele surgery" OR "virtual visit" OR "virtual consultation" OR "virtual care" OR "remote consultation" OR "remote care" OR "remote monitoring" OR facetime OR zoom OR skype OR webex OR "video call" OR videoconference OR "video conference" OR smartphone) AND (anorectal OR anal OR anus OR colorectal OR "colo rectal" OR colon OR rectum OR rectal OR rectosigmoid OR sigmoid OR proctocolonic OR intestine OR colectomy OR hemicolectomy OR ileocolectomy OR proctocolectomy OR stoma OR ileostomy OR enterostomy OR ostomy OR colostomy OR sigmoidectomy OR surgery OR resection) AND (readmission OR "patient satisfaction" OR "emergency visit" OR "emergency admission" OR complication OR infection) AND (colorectal cancer OR rectal cancer OR colon cancer OR colorectal neoplasm OR rectal neoplasm OR colon neoplasm OR tumor OR carcinoma OR "inflammatory bowel disease" OR Crohn OR "ulcerative colitis" OR diverticulitis) |
| 2 | Telemedicine and colorectal surgery |
| 3 | 1 and 2 |

| **Clinicaltrials.gov** | 12-Sep-25 |
| --- | --- |
| (telemedicine OR telehealth OR teleconsultation OR telecare OR telemonitoring OR telerehabilitation OR "tele intervention" OR ehealth OR "tele medicine" OR "tele health" OR "tele communication" OR "tele surgery" OR "virtual visit" OR "virtual consultation" OR "virtual care" OR "remote consultation" OR "remote care" OR "remote monitoring" OR facetime OR zoom OR skype OR webex OR "video call" OR videoconference OR "video conference" OR smartphone) | |
| AND |  |
| (anorectal OR anal OR anus OR colorectal OR "colo rectal" OR colon OR rectum OR rectal OR rectosigmoid OR sigmoid OR proctocolonic OR intestine OR colectomy OR hemicolectomy OR ileocolectomy OR proctocolectomy OR stoma OR ileostomy OR enterostomy OR ostomy OR colostomy OR sigmoidectomy OR surgery OR resection) | |
| AND |  |
| (readmission OR "patient satisfaction" OR "emergency visit" OR "emergency admission" OR complication OR infection) | |
| AND |  |
| (colorectal cancer OR rectal cancer OR colon cancer OR colorectal neoplasm OR rectal neoplasm OR colon neoplasm OR tumor OR carcinoma OR "inflammatory bowel disease" OR Crohn OR ulcerative colitis OR diverticulitis) | |

| **Google Scholar** | |  |
| --- | --- | --- |
| Search date: | | 12-Sep-25 |
| ("telemedicine" OR "telehealth" OR "teleconsultation" OR "telecare" OR "telemonitor*" OR "telerehabilitation" OR "tele-intervention*" OR "ehealth" OR "e-health" OR "tele-medicine" OR "tele-health" OR "tele-communication" OR "tele-surger*" OR "virtual visit*" OR "virtual consultation*" OR "virtual care" OR "remote consultation*" OR "remote care" OR "remote monitoring" OR facetime OR zoom OR skype OR webex OR "video call*" OR videoconference* OR "video conference*" OR smartphone*) | | |
| AND | |  |
| (anorectal OR anal OR anus OR colorectal OR "colo-rectal" OR colon OR rectum OR rectal OR rectosigmoid OR sigmoid OR proctocolonic OR intestin* OR colectom* OR hemicolectom* OR ileocolectom* OR proctocolectom* OR stoma* OR ileostom* OR enterostom* OR ostom* OR colostom* OR sigmoidectom*) | | |
| AND | |  |
| (readmission* OR "patient satisfaction" OR "emergency visit*" OR "emergency admission*" OR "postoperative complication*" OR "post-operative complication*" OR infection*) | | |
| AND | |  |
| ((colon OR rectal OR rectum OR colorectal) AND (neoplasm* OR cancer* OR tumor* OR sarcoma* OR carcinoma*) OR "inflammatory bowel disease" OR "inflammatory bowel disorder" OR diverticulitis) | | |
|  | | |
| **Web of Science** Fri Sep 12 2025 14:21:48 GMT-0400 (Eastern Daylight Time) | | |
| # | Search Query | |
| 1 | TS=(telemedicine OR telehealth OR telecommunication OR telesurger* OR telecare OR teleconsultation* OR teleintervention* OR ehealth OR "tele-medicine" OR "tele-health" OR "tele-communication" OR tele-surger* OR "tele-care" OR "e-health" OR "tele-consultation*" OR "tele-intervention*" OR telerehabilitation OR "tele-rehabilitation" OR ((virtual OR online OR video OR remote OR audio OR phone OR telephone OR distance OR internet OR mobile OR wireless OR chat OR cellphone* OR "cell phone*" OR app OR apps OR application*) NEAR/3 (medicine OR care OR healthcare OR "health care" OR consultation* OR appointment* OR intervention* OR treatment* OR visit*)) OR (facetime OR zoom OR skype OR webex) OR ("video call*" OR videoconference* OR "video conference*" OR smartphone*)) | |
| 2 | TS=(("anorectal" OR "anal" OR "anus" OR "colorectal" OR "colo-rectal" OR "colon" OR "rectum" OR "rectal" OR "rectosigmoid" OR "sigmoid" OR "proctocolonic" OR intestin*) NEAR/3 (surger* OR surgical OR operation* OR procedure* OR resection*) OR colectom* OR hemicolectom* OR ileocolectom* OR proctocolectom* OR stoma* OR ileostom* OR enterostom* OR ostom* OR colostom* OR sigmoidectom*) and Preprint Citation Index (Exclude – Database) and Research Commons (Exclude – Database) | |
| 3 | TS=(readmission* OR ("patient*" NEAR/2 satisf*) OR ((emergency OR ER) NEAR/2 (visit* OR admission* OR consult*)) OR (("post-operative" OR postoperative OR postop OR "post-op") NEAR/2 (complication* OR infection*))) and Preprint Citation Index (Exclude – Database) and Research Commons (Exclude – Database) | |
| 4 | TS=((colon OR rectal OR rectum OR colorectal) NEAR/2 (neoplasm* OR cancer* OR tumo?r* OR sarcoma* OR carcinoma*) OR ("inflammatory bowel" NEAR/2 (disease* OR disorder*)) OR diverticuliti*) and Preprint Citation Index (Exclude – Database) and Research Commons (Exclude – Database) | |
| 5 | #2 OR #4 and Preprint Citation Index (Exclude – Database) and Research Commons (Exclude – Database) | |
| 6 | #5 AND #3 AND #1 and Preprint Citation Index (Exclude – Database) and Research Commons (Exclude – Database) | |

| **Ovid MEDLINE**: Epub Ahead of Print, In-Process & Other Non-Indexed Citations, Ovid MEDLINE® Daily and Ovid MEDLINE® <1946-Present> | |
| --- | --- |
|  |  |
| 1 | exp Telemedicine/ |
| 2 | (telemedicine or telehealth or telecommunication or telesurger* or telecare or teleconsultation* or teleintervention* or ehealth or tele-medicine or tele-health or tele-communication or tele-surger* or tele-care or e-health or tele-consultation* or tele-intervention* or telerehabilitation or tele-rehabilitation).tw,kw,kf. |
| 3 | ((virtual or online or video or remote or audio or phone or telephone or distance or internet or mobile or wireless or chat or cellphone* or cell phone* or app or apps or application*) adj3 (medicine or care or healthcare or health care or consultation* or appointment* or intervention* or treatment* or visit*)).tw,kw,kf. |
| 4 | (facetime or zoom or skype or webex).tw,kw,kf. |
| 5 | (video call* or videoconference* or video conference* or smartphone*).tw,kw,kf. |
| 6 | or/1-5 |
| 7 | colorectal surgery/ |
| 8 | ((anorectal or anal or anus or colorectal or colo-rectal or colon or rectum or rectal or rectosigmoid or sigmoid or proctocolonic or intestin*) adj3 (surger* or surgical or operation* or procedure* or resection*)).tw,kw,kf. |
| 9 | exp Colectomy/ |
| 10 | exp enterostomy/ |
| 11 | (colectom* or hemicolectom* or ileocolectom* or proctocolectom* or stoma* or ileostom* or enterostom* or ostom* or colostom* or sigmoidectom*).tw,kw,kf. |
| 12 | or/7-11 |
| 13 | Patient Readmission/ or exp Postoperative Complications/ or Emergency Service, Hospital/ |
| 14 | Patient Satisfaction/ |
| 15 | readmission*.tw,kw,kf. |
| 16 | (patient* adj2 satisf*).tw,kw,kf. |
| 17 | ((emergency or ER) adj2 (visit* or admission* or consult*)).tw,kw,kf. |
| 18 | ((post-operative or postoperative or postop or post-op) adj2 (complication* or infection*)).tw,kw,kf. |
| 19 | or/13-18 |
| 20 | exp Colorectal Neoplasms/ |
| 21 | ((colon or rectal or rectum or colorectal) adj2 (neoplasm* or cancer* or tumo?r* or sarcoma* or carcinoma*)).tw,kw,kf. |
| 22 | exp Inflammatory Bowel Diseases/ or Diverticulitis/ |
| 23 | (inflammatory bowel adj2 (disease* or disorder*)).tw,kw,kf. |
| 24 | diverticuliti*.tw,kw,kf. |
| 25 | or/20-24 |
| 26 | 12 or 25 |
| 27 | 6 and 19 and 26 |

**Pubmed** (Sep 12, 2025)

((((("Telemedicine"[Mesh] OR telemedicine[tiab] OR telehealth[tiab] OR telecommunication[tiab] OR telesurger*[tiab] OR telecare[tiab] OR teleconsultation*[tiab] OR teleintervention*[tiab] OR ehealth[tiab] OR "tele-medicine"[tiab] OR "tele-health"[tiab] OR "tele-communication"[tiab] OR tele-surger*[tiab] OR "tele-care"[tiab] OR "e-health"[tiab] OR "tele-consultation*"[tiab] OR "tele-intervention*"[tiab] OR telerehabilitation[tiab] OR "tele-rehabilitation"[tiab] OR (("virtual"[tiab] OR online[tiab] OR video[tiab] OR remote[tiab] OR audio[tiab] OR phone[tiab] OR telephone[tiab] OR distance[tiab] OR internet[tiab] OR mobile[tiab] OR wireless[tiab] OR chat[tiab] OR cellphone*[tiab] OR "cell phone*"[tiab] OR app[tiab] OR apps[tiab] OR application*[tiab]) AND (medicine[tiab] OR care[tiab] OR healthcare[tiab] OR "health care"[tiab] OR consultation*[tiab] OR appointment*[tiab] OR intervention*[tiab] OR treatment*[tiab] OR visit*[tiab]))) OR (facetime[tiab] OR zoom[tiab] OR skype[tiab] OR webex[tiab]) OR ("video call*"[tiab] OR videoconference*[tiab] OR "video conference*"[tiab] OR smartphone*[tiab]))) AND (("Colorectal Surgery"[Mesh] OR ((anorectal[tiab] OR anal[tiab] OR anus[tiab] OR colorectal[tiab] OR "colo-rectal"[tiab] OR colon[tiab] OR rectum[tiab] OR rectal[tiab] OR rectosigmoid[tiab] OR sigmoid[tiab] OR proctocolonic[tiab] OR intestin*[tiab]) AND (surger*[tiab] OR surgical[tiab] OR operation*[tiab] OR procedure*[tiab] OR resection*[tiab]))) OR "Colectomy"[Mesh] OR "Enterostomy"[Mesh] OR (colectom*[tiab] OR hemicolectom*[tiab] OR ileocolectom*[tiab] OR proctocolectom*[tiab] OR stoma*[tiab] OR ileostom*[tiab] OR enterostom*[tiab] OR ostom*[tiab] OR colostom*[tiab] OR sigmoidectom*[tiab])))) AND (("Patient Readmission"[Mesh] OR "Postoperative Complications"[Mesh] OR "Emergency Service, Hospital"[Mesh] OR "Patient Satisfaction"[Mesh] OR readmission*[tiab] OR (patient*[tiab] AND satisf*[tiab]) OR ((emergency[tiab] OR ER[tiab]) AND (visit*[tiab] OR admission*[tiab] OR consult*[tiab])) OR (("post-operative"[tiab] OR postoperative[tiab] OR postop[tiab] OR "post-op"[tiab]) AND (complication*[tiab] OR infection*[tiab]))) ))) AND (("Colorectal Neoplasms"[Mesh] OR ((colon[tiab] OR rectal[tiab] OR rectum[tiab] OR colorectal[tiab]) AND (neoplasm*[tiab] OR cancer*[tiab] OR tumo?r*[tiab] OR sarcoma*[tiab] OR carcinoma*[tiab]))) OR ("Inflammatory Bowel Diseases"[Mesh] OR "Diverticulitis"[Mesh] OR (inflammatory bowel[tiab] AND (disease*[tiab] OR disorder*[tiab])) OR diverticuliti*[tiab]) )) ) NOT Medline[sb]

| **Embase** Classic+Embase <1947 to 2025 September 10> | |
| --- | --- |
|  |  |
| 1 | exp telemedicine/ |
| 2 | (telemedicine or telehealth or telecommunication or telesurger* or telecare or teleconsultation* or teleintervention* or ehealth or tele-medicine or tele-health or tele-communication or tele-surger* or tele-care or e-health or tele-consultation* or tele-intervention* or telerehabilitation or tele-rehabilitation).ti,ab,kw. |
| 3 | ((virtual or online or video or remote or audio or phone or telephone or distance or internet or mobile or wireless or chat or cellphone* or cell phone* or app or apps or application*) adj3 (medicine or care or healthcare or health care or consultation* or appointment* or intervention* or treatment* or visit*)).ti,ab,kw. |
| 4 | (facetime or zoom or skype or webex).ti,ab,kw. |
| 5 | (video call* or videoconference* or video conference* or smartphone*).ti,ab,kw. |
| 6 | or/1-5 |
| 7 | exp colorectal surgery/ |
| 8 | ((anorectal or anal or anus or colorectal or colo-rectal or colon or rectum or rectal or rectosigmoid or sigmoid or proctocolonic or intestin*) adj3 (surger* or surgical or operation* or procedure* or resection*)).ti,ab,kw. |
| 9 | exp colectomy/ |
| 10 | exp enterostomy/ |
| 11 | (colectom* or hemicolectom* or ileocolectom* or proctocolectom* or stoma* or ileostom* or enterostom* or ostom* or colostom* or sigmoidectom*).ti,ab,kw. |
| 12 | or/7-11 |
| 13 | 'patient readmission'/ or 'postoperative complication'/ or 'emergency health service'/ |
| 14 | 'patient satisfaction'/ |
| 15 | readmission*.ti,ab,kw. |
| 16 | (patient* adj2 satisf*).ti,ab,kw. |
| 17 | ((emergency or ER) adj2 (visit* or admission* or consult*)).ti,ab,kw. |
| 18 | ((post-operative or postoperative or postop or post-op) adj2 (complication* or infection*)).ti,ab,kw. |
| 19 | or/13-18 |
| 20 | exp colorectal cancer/ |
| 21 | ((colon or rectal or rectum or colorectal) adj2 (neoplasm* or cancer* or tumo?r* or sarcoma* or carcinoma*)).ti,ab,kw. |
| 22 | exp inflammatory bowel disease/ or exp diverticulitis/ |
| 23 | (inflammatory bowel adj2 (disease* or disorder*)).ti,ab,kw. |
| 24 | diverticuliti*.ti,ab,kw. |
| 25 | or/20-24 |
| 26 | 12 or 25 |
| 27 | 6 and 19 and 26 |

| **Central** | |
| --- | --- |
|  | 12/09/2025 19:02:15 |
| #1 |  |
|  | telemedicine OR telehealth OR telecommunication OR telesurger* OR telecare OR teleconsultation* OR teleintervention* OR ehealth OR tele-medicine OR tele-health OR tele-communication OR tele-surger* OR tele-care OR e-health OR tele-consultation* OR tele-intervention* OR telerehabilitation OR tele-rehabilitation |
|  | OR ((virtual OR online OR video OR remote OR audio OR phone OR telephone OR distance OR internet OR mobile OR wireless OR chat OR cellphone* OR cell NEXT phone* OR app OR apps OR application*) NEAR/3 (medicine OR care OR healthcare OR health NEXT care OR consultation* OR appointment* OR intervention* OR treatment* OR visit*)) |
|  | OR (facetime OR zoom OR skype OR webex) |
|  | OR (video NEXT call* OR videoconference* OR video NEXT conference* OR smartphone*) |
|  | ):ti,ab,kw |
| #2 |  |
|  | ((anorectal OR anal OR anus OR colorectal OR colo-next-rectal OR colon OR rectum OR rectal OR rectosigmoid OR sigmoid OR proctocolonic OR intestin*) NEAR/3 (surger* OR surgical OR operation* OR procedure* OR resection*)) |
|  | OR colectom* OR hemicolectom* OR ileocolectom* OR proctocolectom* OR stoma* OR ileostom* OR enterostom* OR ostom* OR colostom* OR sigmoidectom* |
|  | ):ti,ab,kw |
| #3 |  |
|  | readmission* OR (patient* NEAR/2 satisf*) OR ((emergency OR ER) NEAR/2 (visit* OR admission* OR consult*)) OR ((post-operative OR postoperative OR postop OR post-op) NEAR/2 (complication* OR infection*)) |
|  | ):ti,ab,kw |
| #4 |  |
|  | ((colon OR rectal OR rectum OR colorectal) NEAR/2 (neoplasm* OR cancer* OR tumo?r* OR sarcoma* OR carcinoma*)) |
|  | OR (inflammatory NEXT bowel NEAR/2 (disease* OR disorder*)) |
|  | OR diverticuliti* |
|  | ):ti,ab,kw |
| #5 | #1 AND #2 AND #3 AND #4 |

|  | **CINAHL** Fri, September 12, 2025 2:14:50 PM |
| --- | --- |
| **#** | **Query** |
| S6 | S1 AND S3 AND S5 |
| S5 | S2 OR S4 |
| S4 | (MH "Colorectal Neoplasms+") OR ((colon OR rectal OR rectum OR colorectal) N2 (neoplasm* OR cancer* OR tumo?r* OR sarcoma* OR carcinoma*)):TI,AB,SU OR (MH "Inflammatory Bowel Diseases+") OR (MH "Diverticulitis+") OR (inflammatory bowel N2 (disease* OR disorder*)):TI,AB,SU OR diverticuliti*:TI,AB,SU |
| S3 | (MH "Patient Readmission+") OR (MH "Postoperative Complications+") OR (MH "Emergency Service, Hospital") OR (MH "Patient Satisfaction+") OR readmission*:TI,AB,SU OR (patient* N2 satisf*):TI,AB,SU OR ((emergency OR ER) N2 (visit* OR admission* OR consult*)):TI,AB,SU OR ((post-operative OR postoperative OR postop OR post-op) N2 (complication* OR infection*)):TI,AB,SU |
| S2 | (MH "Colorectal Surgery+") OR ((anorectal OR anal OR anus OR colorectal OR "colo-rectal" OR colon OR rectum OR rectal OR rectosigmoid OR sigmoid OR proctocolonic OR intestin*) N3 (surger* OR surgical OR operation* OR procedure* OR resection*)):TI,AB,SU OR (MH "Colectomy+") OR (MH "Enterostomy+") OR (colectom* OR hemicolectom* OR ileocolectom* OR proctocolectom* OR stoma* OR ileostom* OR enterostom* OR ostom* OR colostom* OR sigmoidectom*):TI,AB,SU |
| S1 | (MH "Telemedicine+") OR (TI telemedicine OR AB telemedicine OR SU telemedicine OR TI telehealth OR AB telehealth OR SU telehealth OR TI telecommunication OR AB telecommunication OR SU telecommunication OR TI telesurger* OR AB telesurger* OR SU telesurger* OR TI telecare OR AB telecare OR SU telecare OR TI teleconsultation* OR AB teleconsultation* OR SU teleconsultation* OR TI teleintervention* OR AB teleintervention* OR SU teleintervention* OR TI ehealth OR AB ehealth OR SU ehealth OR TI "tele-medicine" OR AB "tele-medicine" OR SU "tele-medicine" OR TI "tele-health" OR AB "tele-health" OR SU "tele-health" OR TI "tele-communication" OR AB "tele-communication" OR SU "tele-communication" OR TI "tele-surger*" OR AB "tele-surger*" OR SU "tele-surger*" OR TI "tele-care" OR AB "tele-care" OR SU "tele-care" OR TI "e-health" OR AB "e-health" OR SU "e-health" OR TI "tele-consultation*" OR AB "tele-consultation*" OR SU "tele-consultation*" OR TI "tele-intervention*" OR AB "tele-intervention*" OR SU "tele-intervention*" OR TI telerehabilitation OR AB telerehabilitation OR SU telerehabilitation OR TI "tele-rehabilitation" OR AB "tele-rehabilitation" OR SU "tele-rehabilitation") OR ((virtual OR online OR video OR remote OR audio OR phone OR telephone OR distance OR internet OR mobile OR wireless OR chat OR cellphone* OR "cell phone*" OR app OR apps OR application*) N3 (medicine OR care OR healthcare OR "health care" OR consultation* OR appointment* OR intervention* OR treatment* OR visit*)):TI,AB,SU OR (facetime OR zoom OR skype OR webex):TI,AB,SU OR (video call* OR videoconference* OR video conference* OR smartphone*):TI,AB,SU |

| eTable 2. Excluded studies details | | | |
| --- | --- | --- | --- |
| **Study ID** | **Citation (or link if unpublished)** | **Brief rationale** | **Detailed rationale** |
| Amo 2016 | Amo, Leticia; Gonzalez-Lama, Yago; Suarez, Cristina; Blazquez, Isabel; Matallana, Virginia; Calvo, Marta; de la Revilla, Juan; Vera, Maria I.; Abreu, Luis. [Impact of the incorporation of a nurse in an inflammatory bowel disease unit]. Impacto de la incorporacion de la enfermera a una unidad de enfermedad inflamatoria intestinal. 2016;39(5):318-23 | Wrong intervention | Intervention not applied after surgery |
| Ayyoubzadeh 2023 | Ayyoubzadeh SM, Baniasadi T, Shirkhoda M, Rostam Niakan Kalhori S, Mohammadzadeh N, Roudini K, Ghalehtaki R, Memari F, Jalaeefar A Remote Monitoring of Colorectal Cancer Survivors Using a Smartphone App and Internet of Things–Based Device: Development and Usability Study. JMIR Cancer 2023;9:e42250 | Wrong outcomes | Describes development of technology, but none of our included outcomes |
| Bednarzki 2018 | The American Society of Colon and Rectal Surgeon’s Annual Meeting Abstracts. Diseases of the Colon & Rectum 61(5):p e38-e333, May 2018. | Abstract only | Published as an abstract only |
| Bednarzki 2018b | Bednarski, Brian K. M.D.1; Slack, Rebecca S. M.S.2; Katz, Matthew M.D.1; You, Y. Nancy M.D.1; Papadopolous, John M.D.3; Rodriguez-Bigas, Miguel A. M.D.1; Skibber, John M. M.D.1; Matin, Surena F. M.D.3; Chang, George J. M.D.1. Assessment of Ileostomy Output Using Telemedicine: A Feasibility Trial. Diseases of the Colon & Rectum 61(1):p 77-83, January 2018. | Wrong intervention | Intervention occurred in hospital only |
| Bednarzki 2019 | B K Bednarski, T P Nickerson, Y N You, C A Messick, B Speer, V Gottumukkala, M Manandhar, M Weldon, E M Dean, W Qiao, X Wang, G J Chang, Randomized clinical trial of accelerated enhanced recovery after minimally invasive colorectal cancer surgery (RecoverMI trial), BJS, Volume 106, Issue 10, September 2019, Pages 1311–1318, | Wrong intervention | Co-intervention of same day hospital discharge was applied unequally |
| Bertocchi 2021 | Bertocchi E, Barugola G, Gentile I, et aliColon, a patient-focused mobile application for perioperative care in colorectal surgery: an observational, real-world study protocolBMJ Open 2021;11:e045526 | Wrong comparison | Protocol for a study without a comparison group |
| Bertocchi 2023 | Bertocchi E, Barugola G, Masini G, et al. iColon, a patient-focused mobile application for perioperative care in colorectal surgery: Results from 444 patients. Journal of Telemedicine and Telecare. 2023;31(4):585-591. | Wrong comparison | No comparison group |
| Bertocchi 2025a | Bertocchi, E., Masini, G., Barugola, G. et al. ERAS and minimally invasive colorectal surgery: the archetypal non-adherent patients. Surg Endosc 39, 6393–6405 (2025). | Wrong comparison | No comparison group |
| Bertocchi 2025b | Bertocchi E, Barugola G, Masini G, et al. iColon, a patient-focused mobile application for perioperative care in colorectal surgery: Results from 444 patients. Journal of Telemedicine and Telecare. 2023;31(4):585-591. | Wrong comparison | No comparison group |
| Bragg 2017 | Bragg DD, Edis H, Clark S, Parsons SL, Perumpalath B, Lobo DN, Maxwell-Armstrong CA. Development of a telehealth monitoring service after colorectal surgery: A feasibility study. World J Gastrointest Surg 2017; | Wrong comparison | No comparison group |
| Carrier 2016 | Carrier, G.; Cotte, E.; Beyer-Berjot, L.; Faucheron, J. L.; Joris, J.; Slim, K.; Groupe Francophone de Rehabilitation Amelioree apres, Chirurgie. Post-discharge follow-up using text messaging within an enhanced recovery program after colorectal surgery. Journal of visceral surgery 2016;153(4):249-52 | Wrong comparison | No comparison group |
| Curfman 2022 | Curfman KR, Poola AS, Blair GE, Kosnik CL, Pille SA, Thilo EL, Hawkins ME, Rashidi L. Ambulatory colectomy: A pilot protocol for same day discharge in minimally invasive colorectal surgery. Am J Surg. 2022 Aug;224(2):757-760. | Wrong comparison | No comparison group |
| denBakker 2023 | den Bakker, Chantal M; Schaafsma, Frederieke G; Consten, Esther C J; Schraffordt Koops, Steven E; van der Meij, Eva; van de Ven, Peter M; Bonjer, Hendrik J; Huirne, Judith A F; Anema, Johannes R. Personalised electronic health programme for recovery after major abdominal surgery: a multicentre, single-blind, randomised, placebo-controlled trial. The Lancet. Digital health / 2023;5(8):e485-e494 | Wrong patient population | <90% of analyzeable population were colorectal surgery |
| Diehl 2022 | Diehl TM, Barrett JR, Van Doorn R, Cherney Stafford LM, Hanlon BM, Weber SM, Voils CI, Abbott DE. Promoting patient engagement during care transitions after surgery using mobile technology: Lessons learned from the MobiMD pilot study. Surgery. 2022 Jul;172(1):219-225. doi: 10.1016/j.surg.2021.12.018. Epub 2022 Jan 24. | Wrong study design | Pilot study without relevant outcomes |
| Fearn 2019 | Fearn, R. A PATIENT-CENTERED REMOTE CARE PATHWAY TO MANAGE POSTOPERATIVE COMPLICATIONS IN OSTOMATES.. .DISEASES OF THE COLON & RECTUM VOLUME 62: 6 (2019) | Abstract only | Abstract-only for a study that was published and assessed in full text form under separate citation, and also excluded for no comparison |
| Fearn 2020 | Fearn, Robert I. M.D., M.R.C.P.1,2; Gorgun, Emre M.D.3; Sapci, Ipek M.D.3; Mehta, Saahil N. M.D.2; Dinh, Binh B.S.2; Yowell, Quinn V. M.S.2; Eisenstein, Samuel M.D.4. Improved 30-Day Surgical Outcomes in Ostomates Using a Remote Monitoring and Care Management Program: An Observational Study. Diseases of the Colon & Rectum 63(12):p e581-e586, December 2020. | Wrong comparison | Compared to published national database (no study comparison group) |
| Grahn 2019 | Grahn, Sarah W. M.D.1; Lowry, Ann C. M.D.1; Osborne, Marc C. M.D.1; Melton, Genevieve B. M.D., Ph.D.2; Gaertner, Wolfgang B. M.D., M.Sc.2; Vogler, Sarah A. M.D., M.B.A.1; Madoff, Robert D. M.D.2; Kwaan, Mary R. M.D., M.P.H.2. System-Wide Improvement for Transitions After Ileostomy Surgery: Can Intensive Monitoring of Protocol Compliance Decrease Readmissions? A Randomized Trial. Diseases of the Colon & Rectum 62(3):p 363-370, March 2019. \| DOI: 10.1097/DCR.0000000000001286 | Wrong intervention | Not postop telemedicine |
| Hao 2023 | Hao, Ju; Xu, Yanyan; Li, Huiyan. The Value of Applying a Continuous Nursing Model Based on Virtual Platforms for Patients with Colostomy or Ileostomy. Advances in skin & wound care / 2023;36(4):206-212 | Wrong outcomes | Measured self-care, anxiety, health and stoma complications only |
| Hsu 2022 | Hsu, Angela Ting-Wei M.D., M.P.H.; Crawford, Todd C. M.D.; Zhou, Xun M.D.; Safar, Bashar M.B.B.S.; Efron, Jonathan M.D.; Atallah, Chady M.D.; Najjar, Peter A. M.D., M.B.A.; Girard, Andrea L. M.S.N.; Glover, Janelle C. P.A.-C.; Warczynski, Tam C.R.N.P.; Cowell, Nicole A. B.S.N.; Cwik, Carol L. B.S.N.; Fang, Sandy H. M.D.. Decreasing Readmissions After Ileostomy Creation Through a Perioperative Quality Improvement Program. Diseases of the Colon & Rectum 65(8):p e797-e804, August 2022 | Wrong comparison | No comparison group |
| Iqbal 2016 | Iqbal A, Raza A, Huang E, Goldstein L, Hughes SJ, Tan SA. Cost Effectiveness of a Novel Attempt to Reduce Readmission after Ileostomy Creation. JSLS. 2017 Jan-Mar;21(1):e2016.00082 | Wrong outcomes | No relevant outcomes |
| Ketelaers 2022 | Ketelaers, Stijn H. J., Nikki van Ham, Kirsten A. A. J. van Pelt, et al. “The Development and Implementation of an Interactive Application for New Ostomy Patients.” Colorectal Disease 25, no. 6 (2023): 1228–37. | Wrong comparison | No comparison group |
| Krouse 2023 | Krouse RS, Zhang S, Wendel CS, Sun V, Grant M, Ercolano E, Hornbrook MC, Cidav Z, Nehemiah A, Rock M, Appel S, Hibbard JH, Holcomb MJ. A randomized prospective trial of an ostomy telehealth intervention for cancer survivors. Cancer. 2024 Feb 15;130(4):618-635. | Wrong patient population | Not immediate post-op patients |
| Kupper 2024 | Kupper BEC, Bernardon EC, Antunes CF, et al. Developing and validation of a smartphone app for post-discharge early follow-up after colorectal cancer surgeries. DIGITAL HEALTH. 2024;10. | Wrong comparison | No comparison group |
| Lee 2022 | Lee, Lawrence MD, PhD*,†; Eustache, Jules MD†; Baldini, Gabriele MD, MSc‡; Liberman, A. Sender MD*; Charlebois, Patrick MD*; Stein, Barry MD*; Fiore, Julio F. Jr PhD†; Feldman, Liane S. MD*,†. Enhanced Recovery 2.0 – Same Day Discharge With Mobile App Follow-up After Minimally Invasive Colorectal Surgery. Annals of Surgery 276(6):p e812-e818, December 2022. | Wrong comparison | No comparison group |
| Leenen 2023 | Leenen J, Ardesch V, Patijn G Remote Home Monitoring of Continuous Vital Sign Measurements by Wearables in Patients Discharged After Colorectal Surgery: Observational Feasibility Study JMIR Perioper Med 2023;6:e45113 | Wrong comparison | No comparison group |
| Limbach 2023 | Limbach, KE; Fan, D; Melstrom, LG. Remote Telemonitoring and Telehealth in Surgical Oncology. In Advances in Oncology 2023 | Wrong study design | This is a textbook chapter |
| Liu 2021 | Liu, Y.; Ni, L. Construction and application of remote continuing care model for colorectal cancer patients in the internet era. Indian journal of pharmaceutical sciences 2021;83():177 | Wrong outcomes | No relevant outcomes |
| Lixin 2020 | Lixin, Song; Idiagbonya, Eno; Kevin, Su; Idiagbonya, Juliet. Development of a Personalized mHealth Program for Cancer Symptom and Complication Management...30th Medical Informatics Europe Conference. Studies in Health Technology & Informatics 2020;270():1255-1256 | Abstract only | Published as an abstract only |
| Mamlin 2025 | Mamlin M, Khamzina S, Zhanmukanbetova G, Mukazhanov N, Amangeldiyeva A and Kozhakhmetov A (2025) The implementation of the mobile application “Colorectal Leakage App”, based on the Dutch Leakage Score, for early detection of anastomotic leakage after colorectal surgeries—pilot study. Front. Surg. 12:1538023 | Wrong comparison | No comparison group |
| Meacham 2020 | Meacham, Kim; Sneider, Jeremy; Pineda, Theresa. THE INs AND OUTs OF CREATING A STREAMLINED SYSTEMATIC OSTOMY EDUCATION PATHWAY FOR COLORECTAL SURGICAL PATIENTS; A SINGLE-CENTER APPROACH...Scientific and Clinical Abstracts From WOCNext 2020 Reimagined, June 5-7, 2020. Journal of Wound, Ostomy & Continence Nursing 2020;47():S44-S44 | Abstract only | Published as an abstract only |
| Mihaljevic 2024 | CHIR-Net SIGMA Study Group; Mihaljevic, Andre L. MD, MSc. Postoperative Complications and Mobilization Following Major Abdominal Surgery With Versus Without Fitness Tracker-based Feedback (EXPELLIARMUS): A Student-led Multicenter Randomized Controlled Clinical Trial of the CHIR-Net SIGMA Study Group. Annals of Surgery 280(2):p 202-211, August 2024. | Wrong patient population | <90% of analyzeable population were colorectal surgery |
| Miller 2020 | Miller, Morven; Roxburgh, Campbell S.; McCann, Lisa; Connaghan, John; Van-Wyk, Hester; McSorley, Stephen; Maguire, Roma. Development of a Remote Monitoring Application to Improve Care and Support Patients in the First 30 Days Following Colorectal Cancer Surgery. Seminars in oncology nursing 2020;36(6):151086 | Wrong study design | Describes the development of an app, not testing the app |
| NCT06269198 | <https://clinicaltrials.gov/study/NCT06269198?term=NCT06269198&rank=1> | Wrong intervention | In hospital intervention where patient vitals are sent to the nurses' smartphones |
| NCT07113717 | <https://clinicaltrials.gov/study/NCT07113717?term=NCT07113717&rank=1> | Wrong comparison | No comparison group |
| Niu 2021 | Niu, N. U.; Zhi, X. X.; Zhang, L. L.; Wang, M. X.; Zhao, Y.; Yang, L. F.; Wu, B.; Zhu, P.; Wang, N.; Zhang, M. M.; Wu, B. N.; Zhang, Y. A.; Meng, A. F.. Development and implementation of an "Internet plus " management plan for cancer patients with wounds/stomas during the coronavirus disease 2019 pandemic. Precis. Med. Sci. 2021;10(4):159-166 | Wrong outcomes | No relevant outcomes |
| Pakarinen 2022 | Pakarinen S, Varpe P, Carpelan A, Koivisto M, Huhtinen H. Mobile-CEA – A Novel Surveillance Method for Patients with Colorectal Cancer. Cancer Control. 2022;29. | Wrong intervention | Colon cancer surveillance application |
| Rioja 2025 | Rioja, P., Ruiz, R., Araujo, J. et al. e-PRO within a Comprehensive Companion Program to reduce emergency visits and inpatient admission in a Peruvian institution. Support Care Cancer 33, 372 (2025) | Wrong patient population | <90% of analyzeable population were colorectal surgery |
| Symer 2017a | Symer, M.; Abelson, J. S.; McClure, B.; Yeo, H. Pilot study of a novel mobile health application to track patients after GI surgery. Gastroenterology 2017;152(5):S1213 | Abstract only | Published as an abstract only |
| Symer 2017b | Symer, Matthew; Abelson, Jonathan; Milsom, Jeffrey; McClure, Bridget; Yeo, Heather; Symer, Matthew M.; Abelson, Jonathan S.; Yeo, Heather L. A Mobile Health Application to Track Patients After Gastrointestinal Surgery: Results from a Pilot Study. Journal of Gastrointestinal Surgery 2017;21(9):1500-1505. | Wrong comparison | No comparison group |
| VanDerStorm 2023 | S L Van Der Storm, M Jansen, H A Marsman, G P Molly, E C Consten, L M Groot, F C Boer, M Corver, S J Oosterling, J B Tuynman, W A Bemelman, C J Buskens, M P Schijven, P486 Improving enhanced recovery after surgery (ERAS): the effect of a patient-centred mobile application on patient participation in colorectal surgery, Journal of Crohn's and Colitis, Volume 17, Issue Supplement_1, February 2023, Pages i616–i617 | Abstract only | This is an abstract - The full text was retried under separate citation and included in the final analysis |
| Wu 2021 | Wu J; Wei C; Li F; Wang X; Sun F. The effect of comprehensive nursing on the recovery speed and prognosis of elderly colon cancer patients. Am J Transl Res 2021;13(5):5491-5497 | Wrong intervention | No telemedicine/ehealth intervention used |
| Xu 2023a | Xu L, Zhou MZ. Effect of internet multiple linkage mode-based extended care combined with in-hospital comfort care on colorectal cancer patients undergoing colostomy. World J Gastrointest Surg 2023; 15(9): 1959-1968 | Wrong outcomes | No included outcomes reported |
| Xu 2023b | Xu, S., Tan, X., Ma, C. et al. An eHealth symptom and complication management program for cancer patients with newly created ostomies and their caregivers (Alliance): a pilot feasibility randomized trial. BMC Cancer 23, 532 (2023). | Wrong patient population | <90% of analyzeable population were colorectal surgery |
| Yang 2024 | Yang J.; Carmichael J.; Whealon M.; Nielsen T.; Whealon C. PILOT WOCN TELEPHONE FOLLOW UP PROGRAM FOR NEW ADULT OSTOMATES AFTER COLORECTAL SURGERY. Diseases of the Colon and Rectum / 2024;67(6):e1368 | Abstract only | Published as an abstract only. No full text publication available |

| eTable 3. Detailed Characteristics of Ongoing studies | | | |
| --- | --- | --- | --- |
| 1 | **General characteristics** | **Study_id** | Diehl 2022 |
|  |  | **Link** | <https://doi.org/10.1016/j.cct.2021.106658> |
|  |  | **Study contact** | Corrine I. Voils: voils@surgery.wisc.edu |
|  |  | **Country** | USA |
|  |  | **Study_period** | October 2020 to February 2022 |
|  |  | **Study_type** | Open label parallel group RCT |
|  |  | **Funding** | Government/non-profit funding |
|  |  | **Funder_name** | Association of VA Surgeons (MSN225549), National Cancer Institute and Fogarty International Center and National Institute of Mental Health of the National Institutes of Health (award numbers T32CA090217 and D43TW010543). The project described was supported by the Clinical and Translational Science Award (CTSA) program, through the NIH National Center for Advancing Translational Sciences (NCATS), grant UL1TR002373 |
|  |  | **Other COI** | None |
|  |  | **COI_details** | NA |
|  |  | **Study_sites** | University of Wisconsin (UW) Hospital and Clinics (Madison, WI) |
|  | **Inclusion criteria** | **Inclusion_criteria** | Age > 18 years, Patient undergoing complex abdominal surgery in the department of Surgical Oncology, Colorectal Surgery, or Transplant Surgery. English speaking. Able to pass a cognitive screening test Access to a smart device capable of running the MobiMD app (tablet or smartphone). Willingness to comply with all study procedures and availability for the duration of study |
|  |  | **Exclusion_criteria** | NOS |
|  | **Intervention details** | **Telemedicine type** | Smart phone application (MobiMD) |
|  |  | **Telemedicine details** | • Available postoperatively only • Automated reminders to perform recovery tasks and document clinical symptoms post-operatively • Postoperative symptom questionnaires with manual daily review from study physician - Abnormal results forwarded to patient's care team |
|  |  | **Anticipated sample size** | 300 (150 per arm) |
|  |  | **Comparison details** | Usual postoperative care without the application |
|  | **Relevant outcomes** | **Outcome** | **Outcome_definition** |
|  |  | **30-day readmission rate** | "the proportion of participants readmitted to the hospital within 30 days of surgery" |
|  |  | **90-day readmissions** | Collected by chart review |
|  |  | **30-day emergency department and urgent care visits** | Collected by chart review |
|  |  | **90-day emergency department and urgent care visits** | Collected by chart review |
|  |  | **30 and 90 day complications** | "Complications and their associated severity as measured by the Clavian-Dindo score are reported at 30 and 90 days postoperatively. These scores are determined by the outcome assessor through chart review" |
|  |  | **Total readmission costs** | "The total readmission hospital cost is calculated using UW fiscal data harvested form Clarity (Epic, Verona, WI). Clarity includes direct/indirect and fixed/variable hospital cost and reimbursement for professional fees" |
| 2 | **General characteristics** | **Study_id** | Storm 2024 |
|  |  | **Link** | <https://doi.org/10.1186/s12913-023-10508-5> |
|  |  | **Study contact** | Marianne Storm, marianne.storm@uis.no |
|  |  | **Country** | Norway |
|  |  | **Study_period** | April 25, 2023 to December 30, 2026 |
|  |  | **Study_type** | Open label parallel group RCT |
|  |  | **Funding** | Government/non-profit funding |
|  |  | **Funder_name** | Norwegian Research Council, Foundation Dam, the University of Stavanger, Norway and ARC Wessex |
|  |  | **Other COI** | None |
|  |  | **COI_details** | NA |
|  |  | **Study_sites** | Two University hospitals in Norway |
|  | **Inclusion criteria** | **Inclusion_criteria** | Inpatient with symptomatic heart failure, decompensation symptoms such as dyspnoea at rest, pulmonary congestion (X-ray, oedema, or positive rales on auscultation) and elevated NT-proBNP > 1000 pg/m, surgically treated for cure stage I–III colon or rectal cancer [47] |
|  |  | **Exclusion_criteria** | Waiting list for a heart transplant, Requires LVAD, Life expectancy < 6 months, Severe mental illness or cognitive impairment • Planned discharge to a nursing home, Participation in other intervention studies, Unable to stand independently, Metastatic cancer, Clavien-Dindo Surgical Complication Score > 3a, Life-threatening acute medical emergencies (e.g., myocardial infarction) |
|  | **Intervention details** | **Telemedicine type** | Smartphone/tablet application (eHealth@Hospital-2-Home, Dignio Connected Care) |
|  |  | **Telemedicine details** | Available postoperative only. - Access to HF and CRC health-related educational information. - patient self-reporting of symptoms, health and well- being (with review from specialized heart failure nurse). - secure messaging between patients and hospital- based nurse navigators (when patients’ vital signs abnormal). - monitoring of vital signs using connected technology |
|  |  | **Anticipated sample size** | 240 (120 per arm; subdivided as 180 with heart failure, 60 with colorectal cancer) |
|  |  | **Comparison details** | Routine post-operative care |
|  | **Relevant outcomes** | **Outcome** | **Outcome_definition** |
|  |  | Perceived self-efficacy to self-manage HF and CRC | Self-Efficacy for Managing Chronic Disease |
|  |  | Treatment burden | Patient Experience with Treatment and Self-Management Scale |
|  |  | HRQoL | EuroQol EQ-5D-5L questionnaire |
|  |  | Collaboration with healthcare services | CollaboRATE |
|  |  | Support from health professionals | Constructive support from health professionals |
|  |  | Healthcare utilisation | Patient self-reports of number of visits to the primary healthcare service and/ or the specialist healthcare service |
|  |  | Readmissions at 30 and 90 days | Count of readmission from electronic medical records |
|  |  | Days alive and out of the hospital | Patient self-reports of number of days alive and out of hospital |
|  |  | Self-care behaviour in HF | European Heart Failure Self-Care Behaviour Scale |
|  |  | Readmissions at 30 and 90 days and 6 months | Count of readmission from electronic medical records |
|  |  | Medication adherence | Medication Adherence Reasons Scale-5 |
| 3 | **General characteristics** | **Study_id** | NCT06540430 |
|  |  | **Link** | <https://clinicaltrials.gov/study/NCT06540430?term=NCT06540430&rank=1> |
|  |  | **Study contact** | Name: Sanghyun An, M.D., Ph.D.; Phone Number: 82)10-4782-2818; Email: uldura@yonsei.ac.kr |
|  |  | **Country** | Korea |
|  |  | **Study_period** | September 2024 to February 2026 |
|  |  | **Study_type** | Open label parallel group RCT |
|  |  | **Funding** | Not reported |
|  |  | **Funder_name** | NA |
|  |  | **Other COI** | Not reported |
|  |  | **COI_details** | NA |
|  |  | **Study_sites** | Wonju Severance Christian Hospital |
|  | **Inclusion criteria** | **Inclusion_criteria** | Patients aged 19 years or older Patients who have a colostomy or ileostomy after surgery for colon cancer Patients who do not have difficulty using a smartphone |
|  |  | **Exclusion_criteria** | Patients younger than 19 years Patients aged 80 years or older Patients who do not know how to use a smartphone Patients with cognitive impairments Patients with pre-existing psychiatric disorders Patients who have undergone emergency surgery Patients who already have stoma-related complications |
|  | **Intervention details** | **Telemedicine type** | Smart phone application (Ostocare2024) |
|  |  | **Telemedicine details** | Application allows participants to input details of their ostomy care, and receive input regarding management of their care. Unclear when the app is downloaded sequentially related to surgery. Unclear if there is a direct care-provider communication component |
|  |  | **Anticipated sample size** | 120 (60 per arm) |
|  |  | **Comparison details** | Usual care |
|  | **Relevant outcomes** | **Outcome** | **Outcome_definition** |
|  |  | Peristomal Dermatitis | Evaluated using the DET Score (Discoloration, Erosion, and Tissue Overgrowth) on a scale ranging from 0 to 3. At enrollment, 1 month, and 6 months. |
|  |  | Wound Infection, stoma retraction, bleeding, hernia, and Acute kidney injury | Incidence rate as a percentage of patients affected. enrollment, 1 month, and 6 months. |
|  |  | Readmission Rate | Incidence rate as the percentage of patients readmitted due to stoma-related complications, enrollment, 1 month, and 6 months. |
|  |  | Anxiety/depression | EuroQol-5 Dimension scale, at enrollment, 1 month, and 6 months |
|  |  | Quality of life | Stoma quality-of-life scale, at enrollment, 1 month, and 6 months |
| 4 | **General characteristics** | **Study_id** | NCT06593678 |
|  |  | **Link** | <https://clinicaltrials.gov/study/NCT06593678?term=NCT06593678&rank=1> |
|  |  | **Study contact** | Name: José Manuel Burgos Bragado, MSc, Phone Number: +34 646 207 142, Email: jmburgos@usj.es |
|  |  | **Country** | Spain |
|  |  | **Study_period** | August 2024 to August 2025 |
|  |  | **Study_type** | Open label parallel group RCT |
|  |  | **Funding** | Not reported |
|  |  | **Funder_name** | NA |
|  |  | **Other COI** | Not reported |
|  |  | **COI_details** | NA |
|  |  | **Study_sites** | Hospital Royo Villanova, Zaragoze Spain |
|  | **Inclusion criteria** | **Inclusion_criteria** | Age between 18 and 80 years. Participants who understand Spanish. Patients scheduled for colorectal cancer surgery at Royo Villanova Hospital, Zaragoza, Spain. Patients attending the first consultation in the General and Digestive Surgery Section under the supervision of Dr. Blas, head of surgery at Royo Villanova Hospital, Zaragoza, Spain. Participants with functional independence that allows them to perform walking and pulmonary function tests. Patients with a preoperative assessment score of I, II, or III on the American Society of Anesthesiologists (ASA) scale. Participants who agree to participate and sign the informed consent form |
|  |  | **Exclusion_criteria** | Patients older than 80 years. Patients with a preoperative ASA score of IV. Patients with any injury, pathology, or inflammatory processes that make it impossible to practice exercise. Patients with central and/or peripheral neurological diseases that prevent them from following the rehabilitation program. Patients with unstable cardiac comorbidities such as arrhythmias, high blood pressure, angina pectoris, or other conditions that contraindicate moderate-intensity training. Patients diagnosed with a psychiatric disorder as confirmed by a psychiatrist. Patients without access to mobile internet or a computer with internet at home. Patients who score ≤ 24 on the Mini-Mental State Examination (MMSE). Individuals who are unable to follow oral and written instructions in Spanish. Patients who refuse to participate in the study or who have not signed the informed consent form. |
|  | **Intervention details** | **Telemedicine type** | Digital telerehabilitation program |
|  |  | **Telemedicine details** | Available pre- and postoperatively. Digital telerehabilitation program where participants are provided with exercises to perform, and monitoring before and after colorectal surgery. Unclear communication component |
|  |  | **Anticipated sample size** | 54 |
|  |  | **Comparison details** | Usual care and printed rehabilitation information (instead of digital) |
|  | **Relevant outcomes** | **Outcome** | **Outcome_definition** |
|  |  | Functional capacity on Six minute walk test | maximum distance a patient can walk in six minutes on a flat, straight corridor under controlled conditions |
|  |  | Pulmonary capacity using spirometry | Standard spirometry measurements |
|  |  | Upper limb muscle strength | hand grip test |
|  |  | Low limb muscle strength | sit to stand test |
|  |  | Body composition | bioelectrical impedance analysis and waist to hip ratio |
|  |  | Post-operative complications | Comprehensive complication index |
|  |  | Quality of life | Using the EuroQol-5D instument |
|  |  | Anxiety/depression | using the hospital anxiety and depression scale |
|  |  | Psychological adjustment | using the mental adjustment to cancer scale |
|  |  | Sleep quality | Pittsburgh sleep quality index |
|  |  | Adherence to the tele-rehabilitation program | Percentage of prescribed sessions completed by participants |
|  |  | Statisfaction with the telerehabilitation program | Standardized survey using a 4 point scale |
|  |  | Incremental cost-effectiveness ration | Cost per QALY |
|  |  | quality adjusted life years (QALY) | QALY measured based on the value of health outcomes by combinigng both quantity and quality of life |
| 5 | **General characteristics** | **Study_id** | NCT06157814/STUDY-22-00556 |
|  |  | **Link** | <https://clinicaltrials.gov/study/NCT06157814?term=NCT06157814&rank=1> |
|  |  | **Study contact** | Nihal E Mohamed, Icahn School of Medicine at Mount Sinai |
|  |  | **Country** | USA |
|  |  | **Study_period** | June 2023 to May 2025 |
|  |  | **Study_type** | Open label parallel group RCT |
|  |  | **Funding** | Not reported |
|  |  | **Funder_name** | NA |
|  |  | **Other COI** | Not reported |
|  |  | **COI_details** | NA |
|  |  | **Study_sites** | Icahn School of Medicine at Mount Sinai |
|  | **Inclusion criteria** | **Inclusion_criteria** | Patients undergoing ostomy surgeries for bladder or colorectal cancer; Age 21 or older Physically and mentally able to consent and participate Access to a phone Able to speak/read/write English Has an informal/family caregiver (age 21 or greater) willing to participate in the study |
|  |  | **Exclusion_criteria** | Patients with metastatic cancer, cancer recurrence, or presence of other cancers Not able to speak/read/write English |
|  | **Intervention details** | **Telemedicine type** | Smart phone application (STOMA Care) |
|  |  | **Telemedicine details** | Timing of when the app is used in relation to surgery is unclear. Webpage is accessible on handheld electronic devices, provides education materials. Unclear if there will be a direct communication component with healthcare providers |
|  |  | **Anticipated sample size** | 90 |
|  |  | **Comparison details** | Usual care |
|  | **Relevant outcomes** | **Outcome** | **Outcome_definition** |
|  |  | Number of participants recruited |  |
|  |  | Number of participants retained for entirety of study | |
|  |  | Stoma/ostomy knowledge | Assessed via standardized questionnaire |
|  |  | Illness perception | Assessed via illness perception questionnaire |
|  |  | Perception of self-efficacy | Assessed using standardized scale |
|  |  | Quality of life | Via standardized scales |
|  |  | Number of nurse home visits |  |
|  |  | Number of emergency room visits |  |
|  |  | Symptoms | Via multiple scales/questionnaires |
|  |  | Satisfaction with care/communication | Using a standardized scale (unclear if previously/externally validated) |
| 6 | **General characteristics** | **Study_id** | NCT06302751 |
|  |  | **Link** | <https://clinicaltrials.gov/study/NCT06302751?term=NCT06302751&rank=1> |
|  |  | **Study contact** | Name: Annalisa Maroli, PhD, Phone Number: 02 8224 7776 ext 0039, Email: colorapp@humanitas.it |
|  |  | **Country** | Italy |
|  |  | **Study_period** | February 2024 to August 2026 |
|  |  | **Study_type** | Prospective observational study with historical controls |
|  |  | **Funding** | Not reported |
|  |  | **Funder_name** | NA |
|  |  | **Other COI** | Not reported |
|  |  | **COI_details** | NA |
|  |  | **Study_sites** | Istituto Clinico Humanitas, Italy |
|  | **Inclusion criteria** | **Inclusion_criteria** | Patients aged more than 18 years old. Patients scheduled for elective curative colorectal resection, including right (or extended) colectomy, left colectomy, rectal resection (with or without ileostomy), total colectomy, recanalization, or multiple colon resection. Patients willing to comply with the study procedures. Proficient understanding of the Italian language |
|  |  | **Exclusion_criteria** | Any condition that, in the opinion of the investigator, may interfere with the study procedures. Patients scheduled for palliative surgery. Patients with a planned elective intervention requiring urgent or emergent surgery will be withdrawn from the study. Pregnant or breastfeeding women. Inability to comply with the study procedures. |
|  | **Intervention details** | **Telemedicine type** | Smart phone application (Care 4 Today) |
|  |  | **Telemedicine details** | Available pre- and postoperatively. Provides educational materials. Tracks patient symptoms. Allows patient-provider communication. |
|  |  | **Anticipated sample size** | 600 |
|  |  | **Comparison details** | Historical controls prior to device implementation |
|  | **Relevant outcomes** | **Outcome** | **Outcome_definition** |
|  |  | Unplanned hospital readmissions | within 30 days of surgery |
|  |  | Severity of postoperative complications | Clavien-Dindo scale |
|  |  | Lenth of hospital stay | From surgery to discharge date |
|  |  | Time from symptom onset to diagnosis |  |
|  |  | Healthcare cost |  |
| 7 | **General characteristics** | **Study_id** | NCT06850688 |
|  |  | **Link** | https://clinicaltrials.gov/study/NCT06850688?term=NCT06850688&rank=1 |
|  |  | **Study contact** | Name: Emilie DUCHALAIS, Phone Number: +33 2 40 08 43 22, Email: emilie.duchalais@chu-nantes.fr |
|  |  | **Country** | France |
|  |  | **Study_period** | June 2025 to October 2026 |
|  |  | **Study_type** | Open label parallel group RCT |
|  |  | **Funding** | Not reported |
|  |  | **Funder_name** | NA |
|  |  | **Other COI** | Not reported |
|  |  | **COI_details** | NA |
|  |  | **Study_sites** | 12 Hospitals in France |
|  | **Inclusion criteria** | **Inclusion_criteria** | Man or woman over 18 years old at the time of the J0 visit, Patient who has undergone colorectal surgery not managed on an outpatient basis at the investigator center, At least one of the following two criteria: Anticipated early discharge (within 24 hours following laparoscopic surgery without diversion and within 5 days following surgery by laparotomy with stoma) for a surgery not managed on an outpatient basis in routine practice, Estimated risk of hospital readmission greater than 10% according to the nomogram of Tevis et al. [15], adapted to the postoperative context in digestive surgery (>120 points according to this score). Patient affiliated with a health insurance plan, Patient who has signed the study consent form. |
|  |  | **Exclusion_criteria** | Discharge to a rehabilitation center requested by the patient, Condition managed on an outpatient basis in routine practice for this type of patient, Patient eligible for home hospitalization services, Suicidal or hetero-aggressive risk in a patient living alone at home Homeless patient, Pregnant or breastfeeding women, Patient under legal guardianship, curatorship, or protected legal status, Patient already enrolled in an interventional postoperative follow-up study. |
|  | **Intervention details** | **Telemedicine type** | Smartphone application (EPOCA Intelligence Information System) |
|  |  | **Telemedicine details** | Patient monitoring for 30 days after surgery. Device provides patient monitoring and integrates with other medical devices (e.g., oximeters) |
|  |  | **Anticipated sample size** | 362 |
|  |  | **Comparison details** | Usual care |
|  | **Relevant outcomes** | **Outcome** | **Outcome_definition** |
|  |  | Hospitalization days | Cumulative hospitalization days up to 90 days after surgery |
|  |  | Unplaned hospital readmissions | Readmissions and emergency department visits |
|  |  | Difference between initial hospital discharge date and hospital readmission date | For readmitted patients |
|  |  | Unplanned consultations | within 90 days |
|  |  | Postoperative mobidity | number of patients with a complication |
|  |  | Severe postoperative mobidity | nuber of events with Clavien-Dindo grade 3 or 4 |
|  |  | Pain | assessed on POD 3 and 5 using 10 point VAS |
|  |  | Postoperative ileus | For POD3, and 5 |
|  |  | Return to professional activities | Date of return to professional activities (within 90 days) |
|  |  | Quality of life | Via SF-36 and EQ-5D at 30 days and 90 days |
|  |  | Total cost of care | over 90 days |
|  |  | Patient satisfaction | Likert scale evaluated using 3 statements on a 1 to 5 scale |
|  |  | Number of days between surgeru and resumption of both gaseous and fecal transit | within 90 days |
|  |  | Number of patients maintained at home but discontinuing the use of the remote monitoring platform system before the end of follow-up | |
|  |  | The reason for patients remaining at home after discontinuing the use of the remote monitoring platform system before the end of the follow-up | |
|  |  | Number of patients managed with the remote monitoring platform system | |
|  |  | Duration of use of remote monitoring plateform | |
|  |  | Characteristics of the care provided by the remote monitoring platform system | |
|  |  | Patient and care provider perceptions | Qualitative analysis |
| 8 | **General characteristics** | **Study_id** | Steffens 2024/NCT06212700 |
|  |  | **Link** | <https://doi.org/10.1016/j.soi.2024.100082> |
|  |  | **Study contact** | E-mail address: Daniel.Steffens@health.nsw.gov.au (D. Steffens). |
|  |  | **Country** | Australia |
|  |  | **Study_period** | February 2024 to December 2025 |
|  |  | **Study_type** | Open label parallel group RCT (pilot study) |
|  |  | **Funding** | Internal funding |
|  |  | **Funder_name** | Surgical Outcomes Research Centre (SOuRCe). |
|  |  | **Other COI** | None |
|  |  | **COI_details** | NA |
|  |  | **Study_sites** | Royal Prince Alfred Hospital, Sydney Australia |
|  | **Inclusion criteria** | **Inclusion_criteria** | (i) Adults aged ≥ 18 years scheduled for elective major gastrointestinal cancer surgery, including liver, pancreas, oesophagus, gastric, or colorectal cancer resections with curative intent;. (ii) Consulting with a gastrointestinal cancer surgeon at least 1 week prior to scheduled surgery. |
|  |  | **Exclusion_criteria** | (i) Cognitive impairment rendering the patient unable to provide informed consent; (ii) No access to a smart device (including mobile phone, tablet, laptop or desk computer with camera) or no internet connection. |
|  | **Intervention details** | **Telemedicine type** | Videoconference (MS Teams) |
|  |  | **Telemedicine details** | Available pre-and postoperatively. Preoperative educational interventions regarding surgery and ERAS. Postoperative assessment of patients recovery, and tailored interventions to meet recovery needs. Unclear if patients interact with their care team through the app, or only a research personnel |
|  |  | **Anticipated sample size** | 20 (10 per arm) |
|  |  | **Comparison details** | Usual care |
|  | **Relevant outcomes** | **Outcome** | **Outcome_definition** |
|  |  | **Feasibility** | Number of patients recruited, retention rate, adherence to the planned sessions |
|  |  | **Acceptablility** | Patient and provider acceptibility according to standardized questionnaire |
|  |  | **Complication rates/severity** | within 30 days of surgery according to Clavien-Dindo classification |
|  |  | **Quality of life** | According to EORTC QLQ-C30, QLQ-OG25, QLQ-CR29 pre and post surgery |
|  |  | **Number of days at home** | within 30 and 90 days of surgery. **Includes hospital readmissions data** |
|  |  | **Resource use** | Costs |
|  |  | **Implementation outcomes** | according to RE-AIM framework |

# **eTable 4:** Risk of bias assessments for the outcomes of 30-day readmission and 30-day emergency department visits

**eTable 4a:** ROBINS-I for observational studies (both outcomes, 30-day readmissions and emergency department visits were assessed the same for all included studies)

| **Study** | Bias due to confounding | Bias in classification of interventions | Bias in selection of participants into the analysis | Bias due to deviations from intended interventions | Bias due to missing data | Bias in measurement of outcomes | Bias in selection of the reported results | Overall risk of bias |
| --- | --- | --- | --- | --- | --- | --- | --- | --- |
| Borsuk 2018 | Serious | Moderate | Serious | Low | Low | Low | Serious | Serious |
| Zand 2020 | Serious | Low | Moderate | Low | Low | Low | Moderate | Serious |
| Gleason 2022 | Serious | Low | Serious | Low | Low | Low | Moderate | Serious |
| Stapler 2022 | Moderate | Low | Low | Low | Low | Low | Moderate | Moderate |
| Eustache 2023 | Moderate | Low | Moderate | Low | Low | Low | Moderate | Moderate |
| Huber 2025 | Moderate | Low | Low | Low | Low | Low | Low | Moderate |
| Paradis 2023 | Serious | Low | Low | Low | Low | Serious | Moderate | Serious |

**eTable 4b:** ROB 2.0 tool for RCTs (both outcome 30-day readmissions and emergency department visits were assessed the same for all included studies)

| Author | Bias arising from the randomization process | Bias due to deviations in the intended interventions | Bias due to missing outcome data | Bias in measurement of the outcome | Bias in selection of the reported result | Overall risk of bias |
| --- | --- | --- | --- | --- | --- | --- |
| Young 2013 | Low | Low | Low | Low | Low | Low |
| Pooni 2022 | Low | Low | Low | Low | Low | Low |
| Liao 2023 | Some concerns | Low | Low | Low | High | High |
| Van der Storm 2025 | High | High | High | Low | Low | High |

| eTable 5. Summary of findings  **Summary of findings:** | | | | | | |
| --- | --- | --- | --- | --- | --- | --- |
| **Telemedicine compared to usual care for Colorectal surgery** | | | | | | |
| **Patient or population:** Colorectal surgery  **Setting:**  **Intervention:** Telemedicine  **Comparison:** usual care | | | | | | |
| Outcomes | **Anticipated absolute effects^*^** (95% CI) | | Relative effect (95% CI) | № of participants (studies) | Certainty of the evidence (GRADE) | Comments |
|  | **Risk with usual care** | **Risk with Telemedicine** |  |  |  |  |
| 30-Day Readmissions | 117 per 1,000 | **113 per 1,000** (84 to 151) | **OR 0.96** (0.69 to 1.35) | 7102 (11 studies) | ⨁⨁◯◯ Low^a,b^ | Telemedicine may result in little to no difference in 30-Day Readmissions. |
| 30-day emergency department visits | 132 per 1,000 | **135 per 1,000** (128 to 191) | **OR 1.02** (0.67 to 1.57) | 6694 (8 studies) | ⨁⨁◯◯ Low^a,b^ | Telemedicine may result in little to no difference in 30-day emergency department visits. |
| ***The risk in the intervention group** (and its 95% confidence interval) is based on the assumed risk in the comparison group and the **relative effect** of the intervention (and its 95% CI).  **CI:** confidence interval; **OR:** odds ratio | | | | | | |
| **GRADE Working Group grades of evidence** **High certainty:** we are very confident that the true effect lies close to that of the estimate of the effect. **Moderate certainty:** we are moderately confident in the effect estimate: the true effect is likely to be close to the estimate of the effect, but there is a possibility that it is substantially different. **Low certainty:** our confidence in the effect estimate is limited: the true effect may be substantially different from the estimate of the effect. **Very low certainty:** we have very little confidence in the effect estimate: the true effect is likely to be substantially different from the estimate of effect. | | | | | | |

#### Explanations

a. Downgraded for serious risk of bias (all observational studies were at minimum moderate risk of bias)

b. Downgraded for serious imprecision (wide confidence intervals cross zero)

# Supplementary Forest plots

# 1. 30-day readmissions

### eFigure 1. Subgroup analysis 30-day readmissions stratified by smartphone applications and analogue applications (phone calls and email).

### eFigure 2. Subgroup analysis 30-day readmission stratified by timing of telemedicine intervention

### eFigure 3. Sensitivity analysis 30-day readmission with fixed effects model

### eFigure 4. Sensitivity analysis 30-day readmission with risk ratio reported

### eFigure 5. Sensitivity analysis 30-day readmission with risk difference reported

### eFigure 6. Sensitivity analysis 30-day readmission studies at severe risk of bias excluded.

### eFigure 7. Funnel plot for 30-day readmissions stratified by trial design (randomized controlled trial; RCT versus observational studies).

# 2. 30-day emergency department visits

### eFigure 8. Subgroup analysis 30-day emergency department visits stratified by smartphone applications and analogue applications (phone calls and email).

### eFigure 9. Subgroup analysis 30-day emergency department visits stratified by time of telemedicine intervention

### eFigure 10. Sensitivity analysis 30-day emergency department visits with fixed effects model

### eFigure 11. Sensitivity analysis 30-day emergency department visits with risk ratio reported

### eFigure 12. Sensitivity analysis 30-day emergency department visits with risk difference reported

# 3. Patient satisfaction

### eFigure 13. Sensitivity analysis patient satisfaction with van der Storm 2025 imputed from other studies

### eFigure 14. Sensitivity analysis patient satisfaction imputed van der Storm 2025 standard deviation imputed as a small value

### eFigure 15. Sensitivity analysis patient satisfaction with van der Storm 2025 imputed as a large value

### eFigure 16. Sensitivity analysis patient satisfaction with a fixed effects model

### eFigure 17. Sensitivity analysis patient satisfaction with only RCTs included (Li 2024 excluded)
